# Supplementary figures and images for: Single-cell RNA sequencing of Plasmodium vivax sporozoites reveals stage- and species-specific transcriptomic signatures
Source: PLoS Negl Trop Dis. 2022 Aug 4;16(8):e0010633. doi: 10.1371/journal.pntd.0010633 (PMC9380936; doi:10.1371/journal.pntd.0010633)

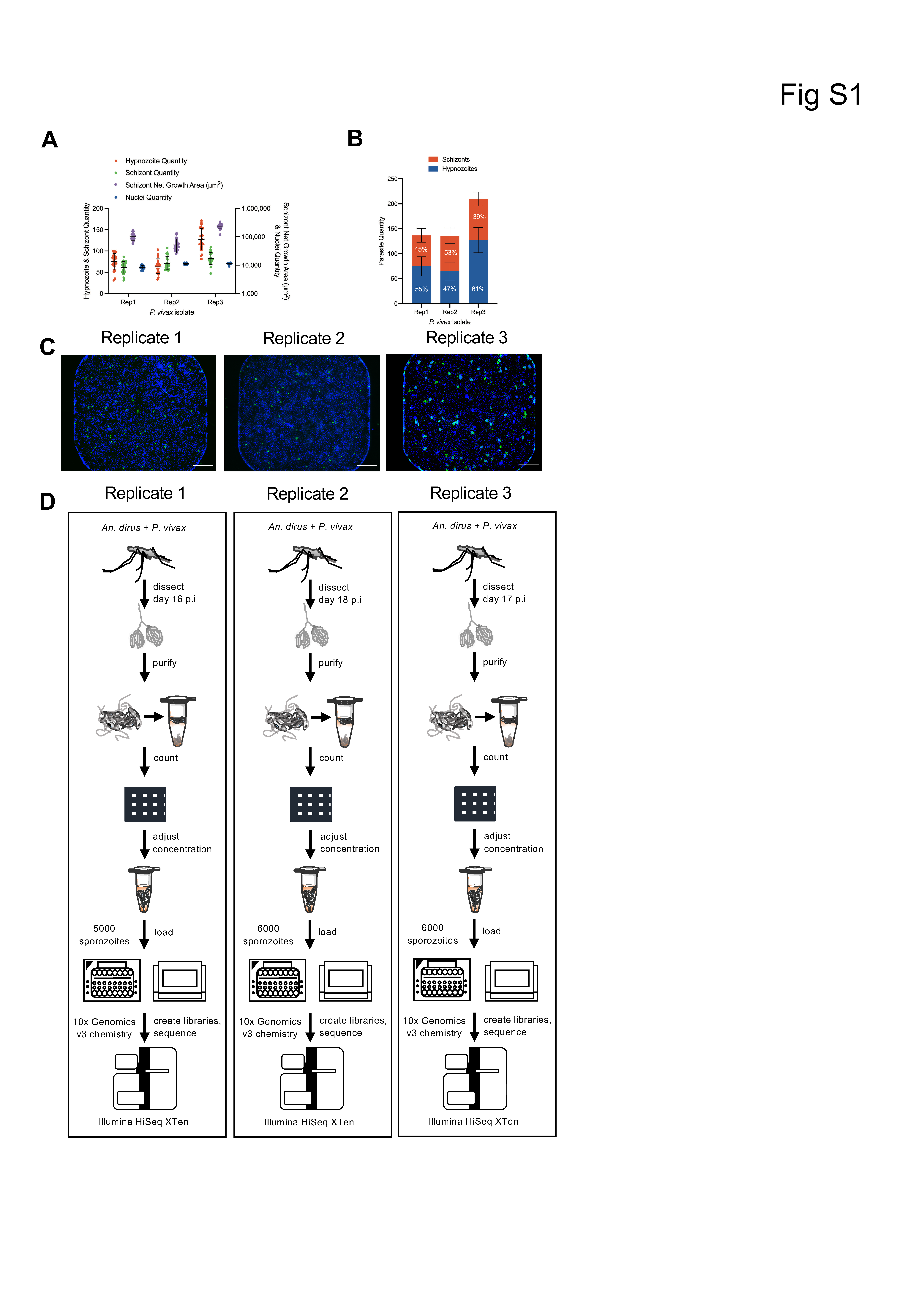

Supplement: S1 Fig — Strategy used to assess P. vivax sporozoite transcriptomes at single-cell resolution. (A) Quantity of all hypnozoites, schizonts, and hepatic nuclei, as well as net growth area of schizonts, following infection of primary human hepatocytes with sporozoites from the indicated P. vivax replicate. Cultures were quantified at 12 days post infection. Each data point represents a single well of 24 technical replicate wells of a 384-well microtiter plate, bars represent SD across wells. The quantity of sporozoites infected into each well was 17,000 for Rep1, 16,500 for Rep2, and 18,000 for Rep3. (B) Ratio of hypnozoites versus schizonts for each isolate from ‘A,’ bars represent SD across 24 technical replicate wells. (C) Image of an individual culture well infected with sporozoites from the indicated isolate at 12 days post infection. Images are stitched from four fields of view taken at low-magnification (4x objective) during high- content imaging. Blue: Hoechst-stained host cell and parasite DNA, green: parasitophorous vacuole membrane detected with immunofluorescent staining with recombinant mouse anti-PvUIS4 antibody. Bar represents 500 μm. (D) Detailed schematic of the workflow for generating P. vivax single-cell RNA sequencing libraries. P. vivax sporozoites were manually dissected and purified by isolated the salivary glands of infected An. dirus mosquitoes. Sporozoites were harvested from three independent infections and three different days post- infectious blood-meal. scRNA-seq libraries were generated for the sporozoites using the 10x Genomics’ 3’ gene expression User Guide. (TIFF) [file pntd.0010633.s015.tiff]

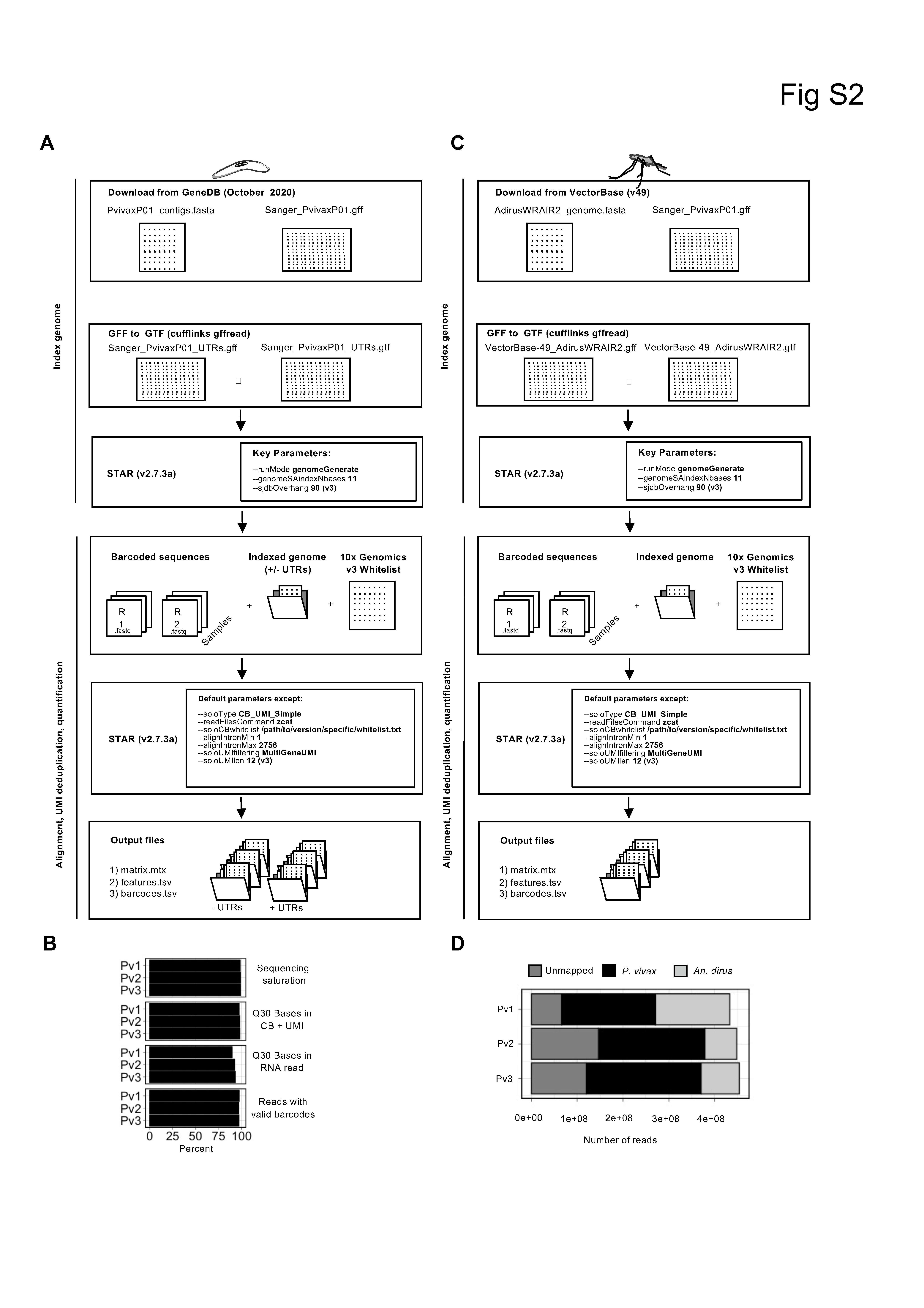

Supplement: S2 Fig — Strategy used to assess P. vivax sporozoite transcriptomes at single-cell resolution. (A) Alignment of reads to P. vivax P01 genome, with parameters listed and output files at each stage. (B) Summary of output metrics from Illumina sequencing. (C) Alignment of reads to An. dirus WRAIR2 genome, with parameters listed and output files at each stage. (D) Number of reads mapping to P. vivax P01 genome, An. dirus WRAIR2 genome or unmapped to either. (TIFF) [file pntd.0010633.s016.tiff]

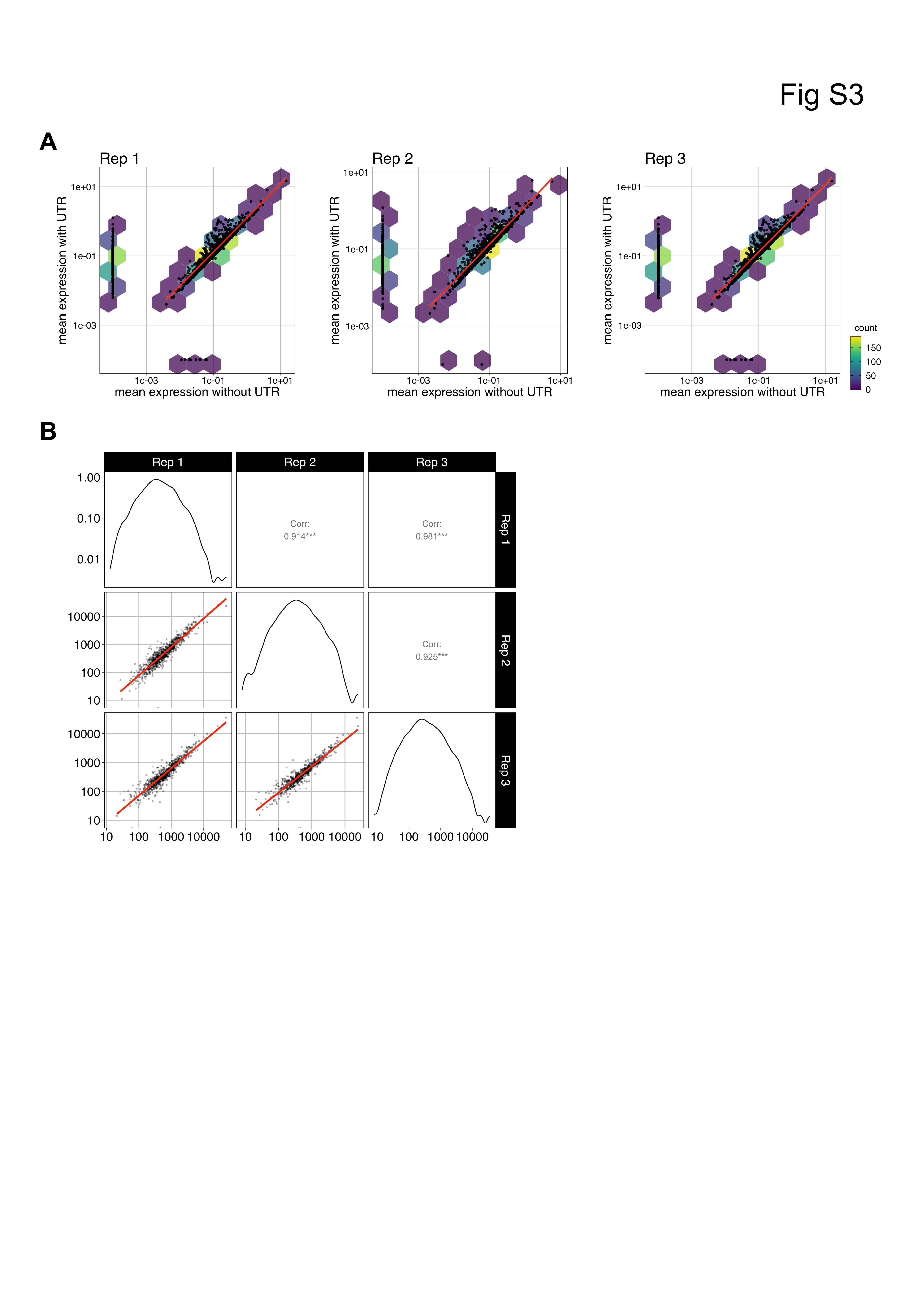

Supplement: S3 Fig — Analysis of P. vivax sporozoite gene expression at single-cell resolution. (A) Pairwise comparisons of transcript abundance (mean expression) with- or without- UTRs in gene models. (B) Pairwise comparisons of transcript abundance (mean expression) across the three replicates when sequencing reads are aligned to the P. vivax P01 genome with UTRs. Pearson’s correlation coefficients (Corr, R) were determined using values > 0 for each pairwise comparison. ***, p value < 0.001. (TIFF) [file pntd.0010633.s017.tiff]

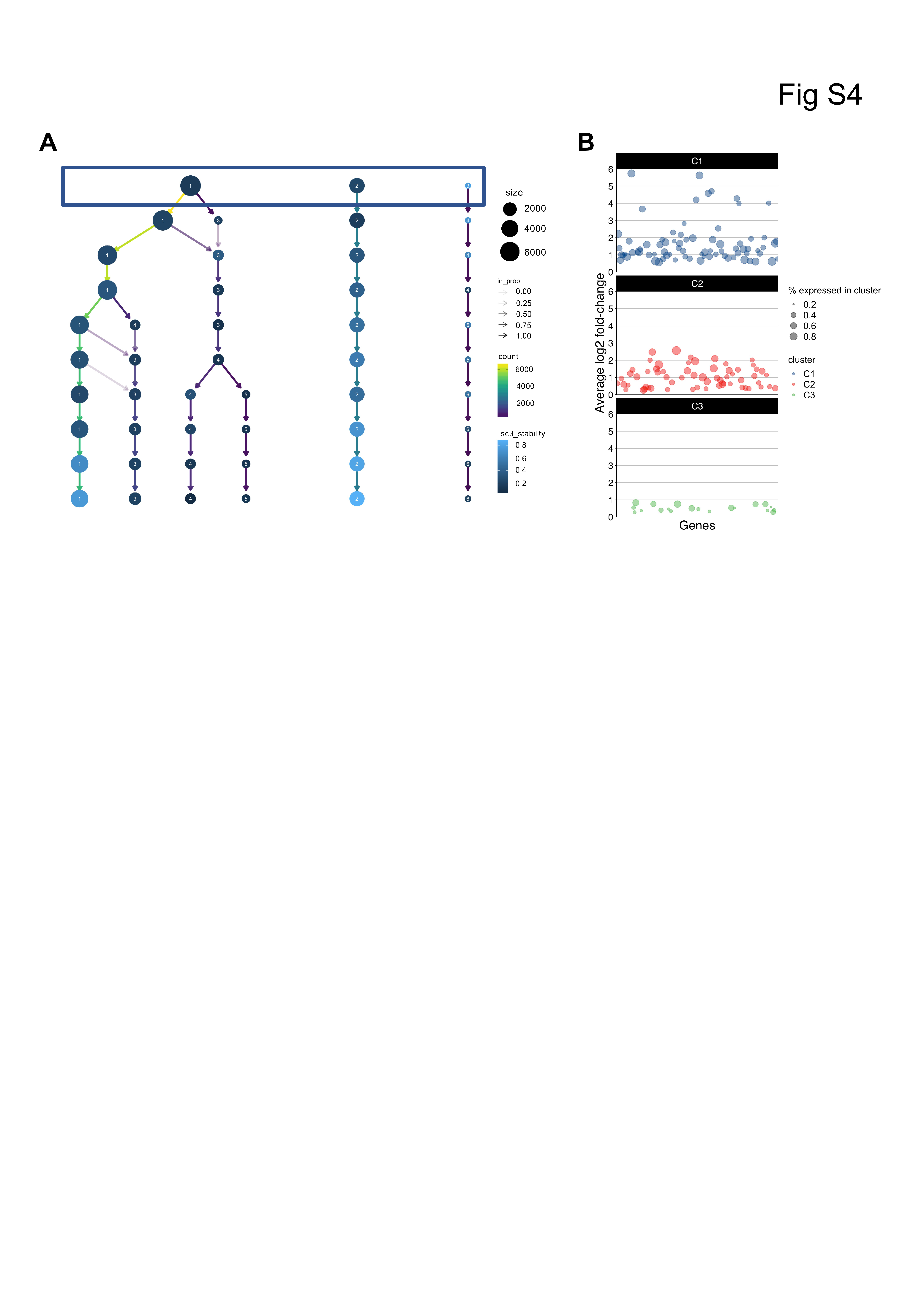

Supplement: S4 Fig — Clustering and differential expression analysis of P. vivax sporozoites. (A) Visualisation of cluster stability when resolution is increased in increments of 0.1 (start = 0.1 and end = 1.0). The size of each point is representative of the number of cells. Edges coloured by number of cells; and transparency represents the incoming node proportion (the number of samples in the edge divided by the number of samples in the node it points to). Point fill (sc3_stability) represents the calculated cluster stability. Clustering tree created with Clustree package (Zappia & Oshlack, 2018). Box indicates clustering resolution used for subsequent differential expression analysis. (B) Scatter plot of 159 genes identified as differentially expressed across the three clusters, split by the cluster. Genes displaying greater expression in each respective cluster plotted. Size of the point represents the percentage of cells expressing the gene of interest. (TIFF) [file pntd.0010633.s018.tiff]

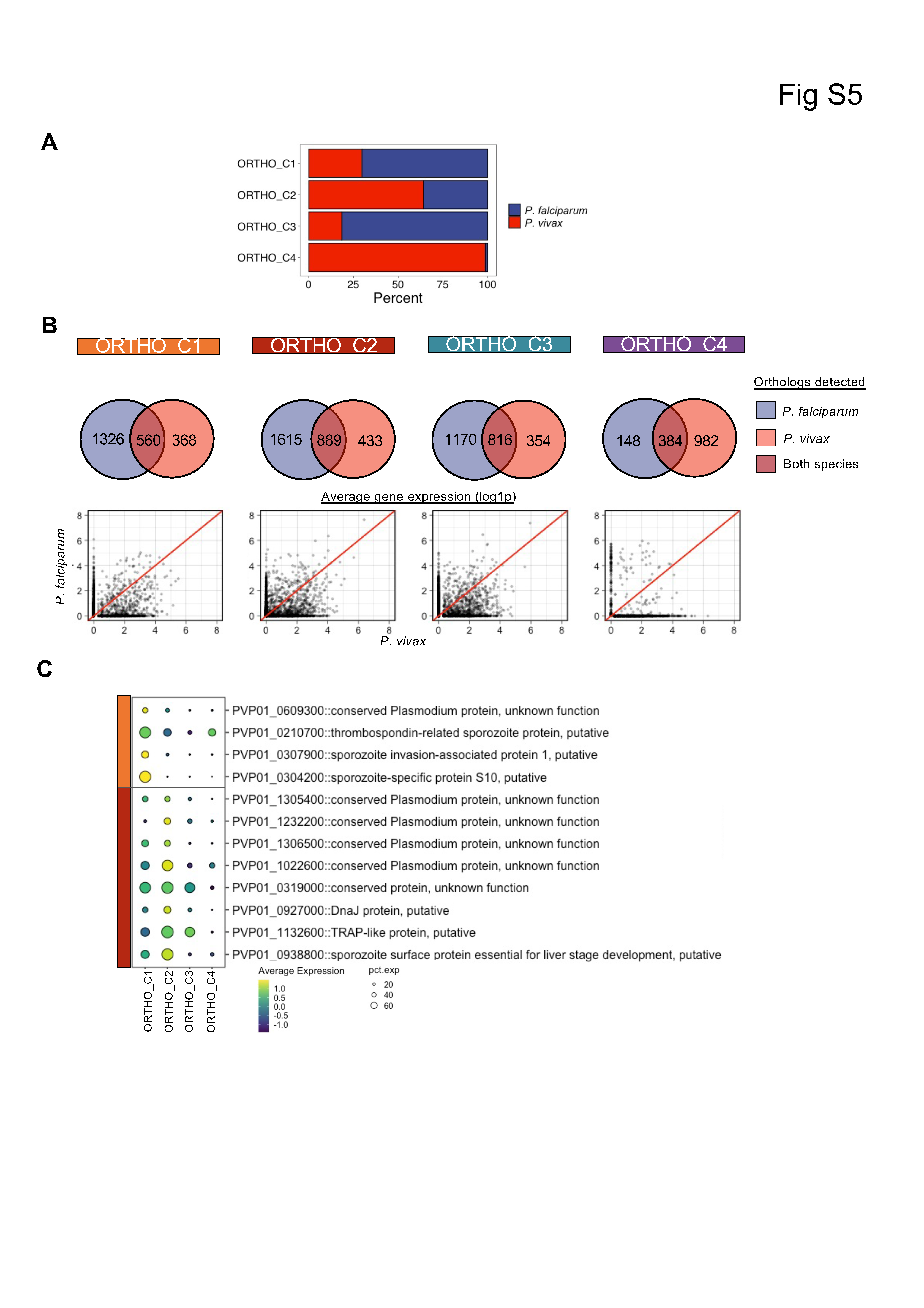

Supplement: S5 Fig — Integration of P. vivax and P. falciparum sporozoite datasets. (A) Proportion of cells derived from P. vivax and P. falciparum in each cluster. (B) Number of overlapping and unique one-to- one orthologs detected in each cluster. (C) Averaged gene expression (log1p) for genes detected in each cluster. (D) Dot plot of the top conserved genes across the two species for clusters one and two (Seurat parameters: min.pct .25, min.diff.pct = 0.125, Wilcoxon rank-sum test). Scale: Normalised expression, scaled; dot size: percentage of cells the transcript is detected. (TIFF) [file pntd.0010633.s019.tiff]

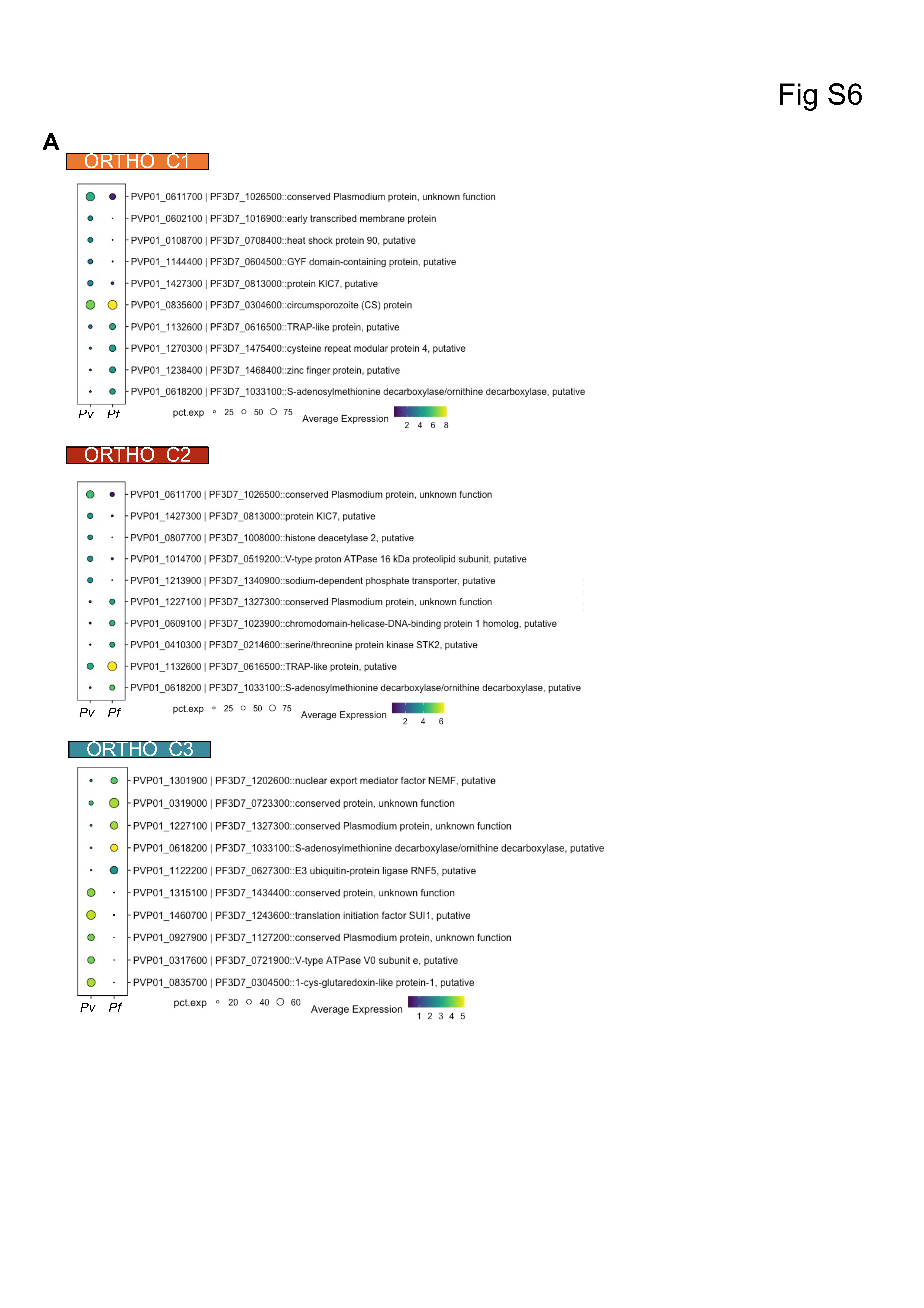

Supplement: S6 Fig — Integration of P. vivax and P. falciparum sporozoite datasets. (A) Dot plots of top one-to-one orthologs in each cluster that are differentially expressed between P. vivax and P. falciparum. The size of the dot corresponds to the percentage of cells expressing the gene. Scale bar: normalised expression. (TIFF) [file pntd.0010633.s020.tiff]

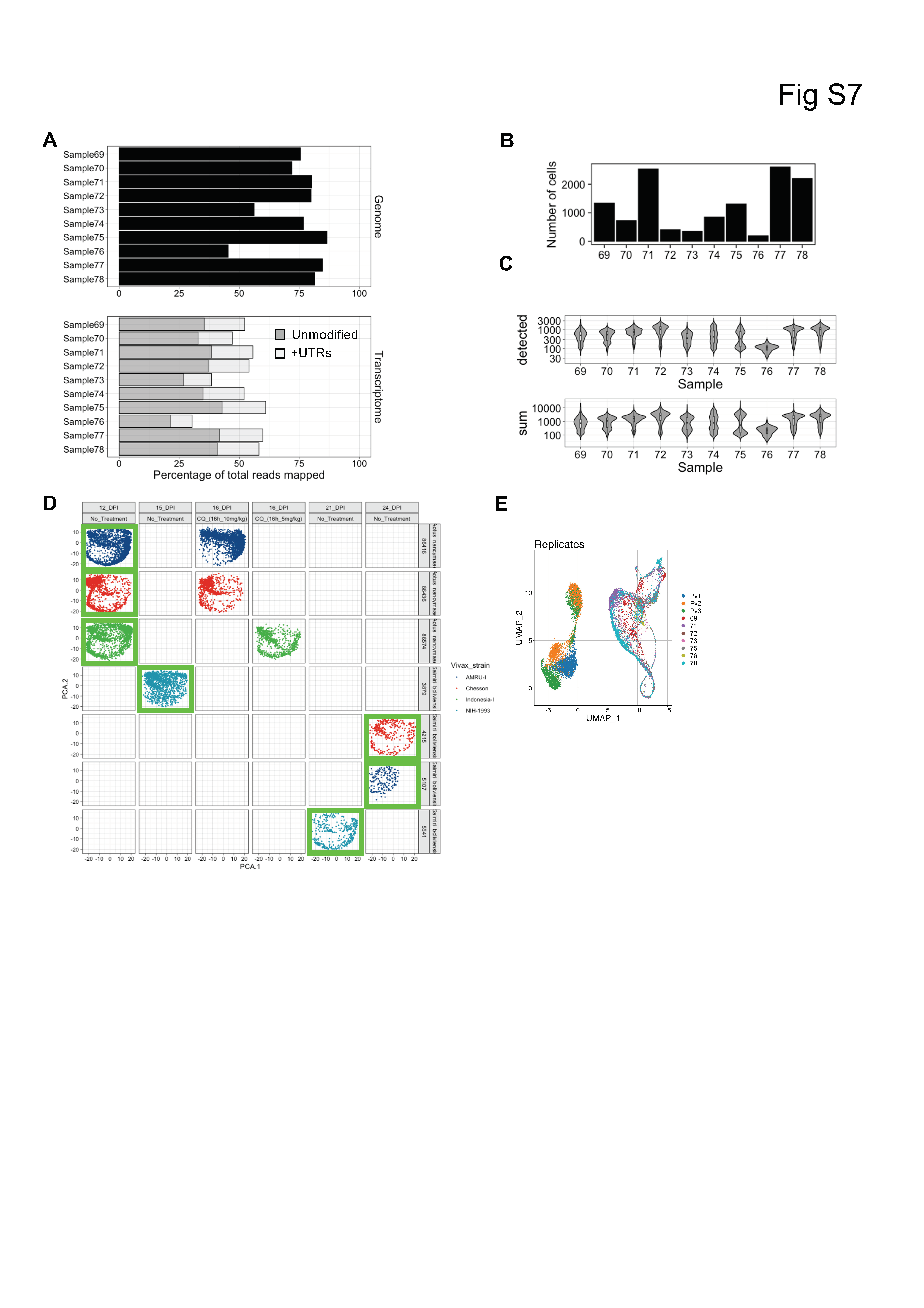

Supplement: S7 Fig — Integration and comparative analyses of P. vivax sporozoite and blood-stage parasite transcriptomes. Realignment, processing, and per-cell metrics of P. vivax blood stage 10x scRNAseq data prior to integration with the P.vivax sporozoite 10x scRNAseq data generated in this study. (A) Percentage of reads aligning to the P. vivax P01 genome (upper panel) and transcriptome (with- or without- UTR information) (lower panel). (B) Number of P. vivax blood-stage transcriptomes retained post cell and gene filtering. (C) Violin plots showing the distribution of genes detected per cell (upper) and the UMIs detected per cell (lower). (D) PCA plots of the samples, split by day of infection, treatment, and monkey. Cells coloured by P. vivax strain used during monkey infection. Samples highlighted in green are those that are integrated with the scRNA-seq sporozoite data of the current study. (E) UMAP of integrated P. vivax sporozoite and blood stage data coloured by sample. (TIFF) [file pntd.0010633.s021.tiff]
