# Supplementary material for: Single-cell RNA sequencing of Plasmodium vivax sporozoites reveals stage- and species-specific transcriptomic signatures
Source: PLoS Negl Trop Dis. 2022 Aug 4;16(8):e0010633. doi: 10.1371/journal.pntd.0010633 (PMC9380936; doi:10.1371/journal.pntd.0010633)
Supplement: Attachment — Submitted filename: PNTD-D-21-01772.pdf [file pntd.0010633.s023.pdf]

PLOS Neglected Tropical Diseases

Single-cell RNA sequencing of Plasmodium vivax sporozoites reveals stage- and species-specific transcriptomic signatures

--Manuscript Draft--

**Manuscript Number:**	PNTD-D-21-01772
**Full Title:**	Single-cell RNA sequencing of Plasmodium vivax sporozoites reveals stage- and species-specific transcriptomic signatures
**Short Title:**	Single-cell RNA sequencing of Plasmodium vivax sporozoites
**Article Type:**	Research Article
**Keywords:**	Plasmodium vivax; single-cell RNA sequencing; sporozoite; malaria; systems biology
**Abstract:**	Background<br>Plasmodium vivax sporozoites reside in the salivary glands of a mosquito before infecting a human host. Previous transcriptome-wide studies in populations of these forms were limited in their ability to elucidate cell-to-cell variation, thereby masking cellular states potentially important in understanding transmission outcomes.<br>Methodology/Principal findings<br>In this study, we performed transcription profiling on 9,947 P. vivax sporozoites to assess the extent to which they differ at single-cell resolution. We show that sporozoites residing in the mosquito's salivary glands exist in distinct developmental states, as defined by their transcriptomic signatures. Additionally, relative to P. falciparum , P. vivax displays overlapping and unique gene usage patterns, highlighting conserved and species-specific gene programs. Notably, distinguishing P. vivax from P. falciparum were a subset of P. vivax sporozoites expressing genes associated with translational regulation and repression. Finally, our comparison of single-cell transcriptomic data from P. vivax sporozoite and erythrocytic forms reveals gene usage patterns unique to sporozoites.<br>Conclusions/Significance<br>In defining the transcriptomic signatures of individual P. vivax sporozoites, our work provides new insights into the factors driving their developmental trajectory and lays the groundwork for a more comprehensive P. vivax cell atlas.
**Additional Information:**	
**Question**	**Response**
**Financial Disclosure**<br><br>Enter a financial disclosure statement that describes the sources of funding for the work included in this submission. Review the submission guidelines for detailed requirements. View published research articles from *PLOS NTDs* for specific examples.<br><br>This statement is required for submission and **will appear in the published article** if the submission is accepted. Please make sure it is accurate.	This work was supported by the Agence Nationale de la Recherche (https://anr.fr, ANR-17-CE13-0025 to A.A.R, G.S., I.M.), the National Health and Medical Research Council of Australia (www.nhmrc.gov.au, NHMRC; 1092789 and 1134989 to I.M.), and a NHMRC Principal Research Fellowship (www.nhmrc.gov.au, 1155075 to I.M). The funders had no role in study design, data collection and analysis, decision to publish, or preparation of the manuscript.

Powered by Editorial Manager® and ProduXion Manager® from Aries Systems Corporation

**Unfunded studies**<br><br>Enter: *The author(s) received no specific funding for this work.*<br><br>**Funded studies**<br><br>Enter a statement with the following details:<br>• Initials of the authors who received each award<br>• Grant numbers awarded to each author<br>• The full name of each funder<br>• URL of each funder website<br>• Did the sponsors or funders play any role in the study design, data collection and analysis, decision to publish, or preparation of the manuscript?<br>• **NO** - Include this sentence at the end of your statement: *The funders had no role in study design, data collection and analysis, decision to publish, or preparation of the manuscript.*<br>• **YES** - Specify the role(s) played.<br><br>* typeset	
**Competing Interests**<br><br>Use the instructions below to enter a competing interest statement for this submission. On behalf of all authors, disclose any competing interests that could be perceived to bias this work—acknowledging all financial support and any other relevant financial or non-financial competing interests.<br><br>This statement **will appear in the published article** if the submission is accepted. Please make sure it is accurate. View published research articles from *PLOS NTDs* for specific examples.	The authors have declared that no competing interests exist.

**NO authors have competing interests**<br><br>Enter: *The authors have declared that no competing interests exist.*<br><br>**Authors with competing interests**<br><br>Enter competing interest details beginning with this statement:<br><br>*I have read the journal's policy and the authors of this manuscript have the following competing interests: [insert competing interests here]*<br><br>* typeset<br><br>This statement is required for submission and **will appear in the published article** if the submission is accepted. Please make sure it is accurate and that any funding sources listed in your Funding Information later in the submission form are also declared in your Financial Disclosure statement.	
**Data Availability**<br><br>Authors are required to make all data underlying the findings described fully available, without restriction, and from the time of publication. PLOS allows rare exceptions to address legal and ethical concerns. See the PLOS Data Policy and FAQ for detailed information.	Yes - all data are fully available without restriction

Powered by Editorial Manager® and ProduXion Manager® from Aries Systems Corporation

A Data Availability Statement describing where the data can be found is required at submission. Your answers to this question constitute the Data Availability Statement and **will be published in the article**, if accepted.<br><br>**Important:** Stating 'data available on request from the author' is not sufficient. If your data are only available upon request, select 'No' for the first question and explain your exceptional situation in the text box.<br><br>Do the authors confirm that all data underlying the findings described in their manuscript are fully available without restriction?	
**Describe where the data may be found in full sentences. If you are copying our sample text, replace any instances of XXX with the appropriate details.**<br><br>• If the data are **held or will be held in a public repository**, include URLs, accession numbers or DOIs. If this information will only be available after acceptance, indicate this by ticking the box below. For example: *All XXX files are available from the XXX database (accession number(s) XXX, XXX.)*.<br>• If the data are all contained **within the manuscript and/or Supporting Information files**, enter the following: *All relevant data are within the manuscript and its Supporting Information files.*<br>• If neither of these applies but you are able to provide **details of access elsewhere**, with or without limitations, please do so. For example:<br><br>*Data cannot be shared publicly because*	All raw sequencing data generated from P. vivax sporozoites in this study will be deposited and accessible in the European Nucleotide Archive (www.ebi.ac.uk/ena/) upon acceptance of the manuscript.

Powered by Editorial Manager® and ProduXion Manager® from Aries Systems Corporation

of [XXX]. Data are available from the XXX Institutional Data Access / Ethics Committee (contact via XXX) for researchers who meet the criteria for access to confidential data.<br><br>*The data underlying the results presented in the study are available from (include the name of the third party and contact information or URL).*<br>• This text is appropriate if the data are owned by a third party and authors do not have permission to share the data.<br><br>* typeset	
Additional data availability information:	Tick here if the URLs/accession numbers/DOIs will be available only after acceptance of the manuscript for publication so that we can ensure their inclusion before publication.

Manuscript

Click here to access/download;Manuscript;PvSpz_scRNAseq_manuscript_PL

Single-cell RNA sequencing of *Plasmodium vivax* sporozoites reveals stage- and species-specific transcriptomic signatures

Short title: Single-cell RNA sequencing of *Plasmodium vivax* sporozoites

Anthony A. Ruberto[1,‡,¤], Caitlin Bourke[2,3, ‡], Amélie Vantaux[4] Steven P. Maher[5], Aaron Jex[2,3], Benoit Witkowski[4], Georges Snounou[6], Ivo Mueller[1,2,3]*

[1]Department of Parasites and Insect Vectors, Institut Pasteur, Paris, France

[2] Division of Population Health and Immunity, Walter and Eliza Hall Institute of Medical Research, Parkville, Victoria, Australia

[3] Department of Medical Biology, The University of Melbourne, Parkville, Victoria Australia

[4] Malaria Molecular Epidemiology Unit, Institut Pasteur du Cambodge, Phnom Penh, Kingdom of Cambodia

[5]Center for Tropical and Emerging Global Diseases, University of Georgia, Athens 30602, USA

[6] Commissariat à l'Énergie Atomique et aux Énergies Alternatives-Université Paris Sud 11-INSERM U1184, Immunology of Viral Infections and Autoimmune Diseases (IMVA-HB), Infectious Disease Models and Innovative Therapies (IDMIT) Department, Institut de Biologie François Jacob (IBFJ), Direction de la Recherche Fondamentale (DRF), Fontenay-aux-Roses, France

‡ AAR and CB contributed equally to this work.

¤ Current address: Center for Tropical and Emerging Global Diseases, University of Georgia, Athens 30602, USA

* Corresponding author

E-mail: mueller@wehi.edu.au

Abstract

Background

Plasmodium vivax sporozoites reside in the salivary glands of a mosquito before infecting a human host. Previous transcriptome-wide studies in populations of these forms were limited in their ability to elucidate cell-to-cell variation, thereby masking cellular states potentially important in understanding transmission outcomes.

Methodology/Principal findings

In this study, we performed transcription profiling on 9,947 *P. vivax* sporozoites to assess the extent to which they differ at single-cell resolution. We show that sporozoites residing in the mosquito's salivary glands exist in distinct developmental states, as defined by their transcriptomic signatures. Additionally, relative to *P. falciparum*, *P. vivax* displays overlapping and unique gene usage patterns, highlighting conserved and species-specific gene programs. Notably, distinguishing *P. vivax* from *P. falciparum* were a subset of *P. vivax* sporozoites expressing genes associated with translational regulation and repression. Finally, our comparison of single-cell transcriptomic data from *P. vivax* sporozoite and erythrocytic forms reveals gene usage patterns unique to sporozoites.

Conclusions/Significance

In defining the transcriptomic signatures of individual *P. vivax* sporozoites, our work provides new insights into the factors driving their developmental trajectory and lays the groundwork for a more comprehensive *P. vivax* cell atlas.

Author summary

Plasmodium vivax is the second most common cause of malaria worldwide. It is particularly challenging for malaria elimination as it forms both active blood-stage infections, as well as asymptomatic liver-stage infections that can persist for extended periods of time. The reactivation of persister forms in the liver (hypnozoites) are responsible for relapsing infections occurring weeks or months following primary infection via a mosquito bite. How *P. vivax* persists in the liver remains a major gap in understanding of this organism. It has been hypothesized that there is pre-programming of the infectious sporozoite while it is in the salivary-glands that determines if the cell’s fate once in the liver is to progress towards immediate liver stage development or persist for long-periods as a hypnozoite. The aim of this study was to see if such differences were distinguishable at the transcript level in salivary-gland sporozoites. While we found significant variation amongst sporozoites, we did not find clear evidence that they are

clearly transcriptionally pre-programmed as has been suggested. Nevertheless, we highlight several intriguing patterns that appear to be *P. vivax* specific relative to non-relapsing species that cause malaria prompting further investigation.

Introduction

Malaria remains the most significant parasitic disease of humans globally, causing an estimated 229 million infections and 409,000 deaths per year [1]. *Plasmodium* spp. are the etiological agents of malaria, and at least five species are known to infect humans [1]. *Plasmodium falciparum* and *P. vivax* are the most prevalent, and both contribute significantly to disease burden [2–4]. *Plasmodium* spp. infection in humans begins with the deposition of sporozoites into the dermis when an infected *Anopheles* mosquito takes a blood meal [5]. While sporozoites must undergo replication in the liver before mounting a blood-stage infection, *P. vivax* sporozoites can develop into either a replicating or persisting (hypnozoite) form [6]. Hypnozoites can remain in the liver for weeks, months or years before reactivating to undergo schizogony [7], leading to a relapsing blood-stage infection. Relapsing infections are estimated to comprise up to 90% of *P. vivax* malaria cases in some regions [8–10]. Relapse-causing hypnozoites, in addition to a high prevalence of sub-detectable and often asymptomatic blood-stage infections, severely limit efforts to eradicate *P. vivax* malaria [3,11]. Recent modelling suggests that eliminating *P. vivax* malaria is not possible without programs that specifically target and cure hypnozoite infections [12].

The factors underlying the development of *P. vivax* sporozoites into replicating schizonts or their persistence as hypnozoites and subsequent reactivation remain poorly defined. Key questions regarding the regulation of hypnozoite biology have focused on how it differs regionally, seasonally, and between strains. Relapse frequency varies by climate and geographical region, with temperate strains exhibiting long periods of latency and tropical ones relapsing at shorter intervals [13–15]. The observation that *P. vivax* may be able to regulate hypnozoite formation in accordance with environmental conditions feeds into a hypothesis that the developmental outcome within the liver may be pre-determined in the sporozoite [16,17]. In addition, observations in humanised rodent livers have identified sympatric *P. vivax* strains with stable differences in hypnozoite formation rates [18]. This points to genetic heterogeneity among *P. vivax* sporozoites that may play a role in defining developmental fate, consistent with the tachy- and bradysporozoites proposed by Lysenko et al. [16]. System-wide studies offer an opportunity to find evidence for sporozoite pre-programming; however, previous analyses of *P. vivax* sporozoites have been performed using bulk-sequencing approaches [19–21] which obscure variation that might exist between individual parasites.

Single-cell RNA sequencing methods (scRNA-seq) constitute a recent advancement applicable for assessing parasite-to-parasite differences. ScRNA-seq has differentiated multiple

transcriptomic states among individual *P. berghei* and *P. falciparum* sporozoites [22–25]. However, the extent to which *P. vivax* sporozoites vary at the single-cell level has not been studied. Therefore, the application of scRNA-seq technology provides an opportunity to explore heterogeneity amongst *P. vivax* sporozoites and examine the existence of distinct transcriptional signatures that may help better understand the sporozoite's developmental fate.

In this study, we analyse the transcriptomes of 9,947 *P. vivax* sporozoites captured using droplet-based scRNA-seq technology. We first cross-reference the data with sporozoite bulk microarray and RNA-seq data to show consistent transcription of known genes upregulated in sporozoites. Next, we represent the data in low dimensional space and identify sporozoites in various transcriptomic states using both clustering and pseudotime trajectory methods. Finally, we perform comparative analyses with publicly available *P. falciparum* sporozoite and *P. vivax* blood-stage scRNA-seq data [23,26] and highlight both conserved and unique gene usage patterns between sporozoites and erythrocytic forms. Overall, our work provides an important, new resource for the malaria community by offering key insights into gene usage among *P. vivax* sporozoites and the factors driving their developmental trajectory at a resolution unattainable with bulk transcriptomics.

Results

Processing, alignment, and pseudo-bulk assessment of *P. vivax* sporozoite scRNA-seq data

Given its high-throughput capability and prior use for sporozoites from murine-infecting *Plasmodium* species [22,25], we used the 10X Genomics gene expression platform to profile the transcriptomes of individual *P. vivax* sporozoites. We dissected and purified sporozoites from the salivary glands of *An. dirus* mosquitoes in triplicate, with each replicate comprising sporozoites derived from mosquitoes fed on a blood-meal from a different patient isolate. Sporozoites dissected and released from mosquito salivary glands were kept in Hank's Balanced Salt Solution at 4°C to minimise their activation. Sporozoites from each replicate were infectious to primary human hepatocytes, as indicated by their ability to generate liver forms in a 384-well microtiter plate platform [27](Figs S1A-C). High-content analysis of liver-stage cultures demonstrated that the sporozoites developed into hypnozoites and schizonts at a ratio of ~4:5 to ~6:4, depending on the case (Fig S1B). Between 5000-8000 sporozoites were loaded on a 10x Genomics' Chromium controller to partition the sporozoites, lyse, and uniquely-tag transcripts. After tagging the transcripts, Illumina compatible short-read libraries were generated and sequenced (Fig 1A and Fig S1D; S1 Table).

After aligning the sequencing data to the *P. vivax* P01 genome (Fig S2A), various metrics confirmed that the libraries were of high quality (Fig S2B). An average of 48% of reads across all replicates (638,820,734/1,340,791,021) mapped to the *P. vivax* P01 genome (Fig 1B), with the remainder mapping to *An. dirus* (32%; 437,925,660/1,340,791,021) or not mapping at all (20%; 264,044,627/1,340,791,021) (Figs S2C and S2D; S2 Table).

Until recently, the undefined nature of untranslated regions (UTRs) in the *P. vivax* transcriptome was a gap in knowledge that restricted accurate quantification of transcription. Given the 10x Genomics' scRNA-seq technology captures specifically the 3' end of transcripts [28–30], RNA with long untranslated regions (UTRs) may be sequenced and mapped to the genome but remain unquantified due to the incomplete annotation of these gene-flanking regions. This is a documented challenge for 3' capture methods in *Plasmodium and* other non-model organisms [22,25,31,32]. However, a more complete *P. vivax* transcriptome, including UTRs, was recently reported [33] and was incorporated into our alignment pipeline (Fig S2A). The inclusion of UTR coordinates resulted in a 1.3-fold increase of reads assigned to *P. vivax* genes (Fig 1B; S3 Table). Compared to the alignment of reads using gene models lacking UTRs, UTR inclusion resulted in the detection of an additional 417 genes and an increase in counts for 1,182 genes (Fig S3A; S2 and S3 Tables). The largest increase was found for the gene encoding gamete egress and sporozoite traversal protein (PVP01_125800) (Fig 1C; S3 Table). Other notable examples of genes with increased transcription included serine threonine protein phosphatase 2B catalytic subunit A (PVP01_0117400), RNA-binding protein Musashi (PVP01_0715100) and a conserved protein of unknown function (PVP01_1011800) (Fig 1C; S3 Table).

We next assessed the transcriptomes of 9,947 sporozoites obtained across all replicates (Fig 1D). Of the 1,387 genes detected, 735 (53%) were detected in all three replicates (Fig 1E). We observed high correlation in the transcription of genes detected in each replicate (mean Pearson correlation coefficient, $R = 0.94$, $p < 0.05$; Fig S3B). Genes encoding for circumsporozoite protein (PVP01_0835600), gamete egress and sporozoite traversal protein (*gest*; PVP01_1258000), and sporozoite protein essential for cell traversal (*spect*; PVP01_1212300) were among those with the highest transcription (S4 Table). Comparative analyses of our data with bulk transcriptomic studies [19,20] revealed consistent detection of various genes implicated in sporozoite biology (Fig 1F). These results paint a clearer picture of gene usage in *P. vivax* sporozoites by incorporating each genes' UTRs and highlight the capacity of scRNA-seq in assessing transcription in *P. vivax* sporozoites.

Assessment of *P. vivax* sporozoites at single-cell resolution reveals transcriptomic heterogeneity

Our assessment of various per-cell metrics revealed differences in *P. vivax* sporozoites at the transcript level, specifically in the distribution of unique molecular identifiers (UMIs); the absolute number of transcripts [34], and genes detected in individual sporozoites across replicates (Fig 2A). Using Uniform Manifold Approximation Projection (UMAP) of the data to assess the transcriptomic differences visually, we found three distinct populations of sporozoites (Fig 2B). Overlaying the UMI and genes detected data on the UMAP revealed sporozoites in two main transcriptomic states: forms with higher gene usage (represented by the cells on the left side of the UMAP) and forms with lower gene usage (on the right) (Fig 2C). We first visualised transcription of well-described sporozoite membrane-associated and cell traversal proteins (Fig 2D) [35–41]. Next, given that *P. vivax* parasites can form both replicating schizonts and hypnozoites in the liver, we assessed the transcription profiles of genes implicated in sporozoite developmental fating [19]. However, no clear pattern was observed, and sporozoites in both populations transcribed genes encoding for membrane proteins, cell traversal and initiation of invasion, and translational repression (Fig 2D).

To better understand the biological significance of these populations, we used an unsupervised graph-based clustering algorithm [42] with a conservative grouping parameter (Fig S4A) to systematically divide the sporozoites into three transcriptional clusters (Fig 3A), consisting of 86 (cluster C1), 6,982 (cluster C2) and 2,879 (cluster C3) sporozoites, respectively. The number of sporozoites in each cluster varied across replicates, indicating their variability across different mosquito infections (Fig 3B). We next identified markers that define the sporozoites in each cluster using the FindAllMarkers function in Seurat [43]. We defined a marker as a gene detected in over 30% of cells in a given cluster and displaying significantly greater transcription than the other clusters. In total, we found 159 markers (adjusted p-value < 0.05, S5 Table). Notably, sporozoite-specific protein, S10 (PVP01_0304200), was a marker for sporozoites in C1 (Fig 3C; S5 Table), and has previously been shown to be highly transcribed in midgut sporozoites [22,25]. C2 markers included circumsporozoite protein (PVP01_0835600); early transcribed membrane protein (PVP01_0602100); TRAP-like protein (PVP01_1132600); and sporozoite surface protein essential for liver stage development (PVP01_0938800) (Fig 3C; S5 Table). Relative to C1 and C2, C3 had the fewest markers (20) (adjusted p-value < 0.05), and of the markers identified, their changes in transcription were modest (average $\log_2$- fold-change range 0.28 – 0.84; Fig S4B). Markers in this cluster included genes involved in proton transport (PVP01_0317600 and PVP01_1117400); redox response (PVP01_0835700 and

PVP01_1249700); and heat shock proteins (PVP01_1440500 and PVP01_1011500) (Fig 3C; S5 Table).

Trajectory-based pseudotime analysis reveals various transcription patterns in sporozoites

A caveat of using a cluster-based classification method is that cells are forced into groups. In developing systems, such as sporozoites, cell transitions may be occurring more continuously. The modest number of markers defining each cluster (S5 Table) suggested that a continuum of transcriptional states may exist. We thus examined the transcriptional profiles of *P. vivax* sporozoites in the context of pseudotime. We used Slingshot [44] to construct a trajectory through the cells (Fig 4A). We observed differences in the distribution of sporozoites among replicates over pseudotime. Sporozoites from replicate 1 were unimodal and primarily enriched earlier in the trajectory, whereas sporozoites from replicates 2 and 3 were bimodal, with more cells near the end of the trajectory (Fig 4B).

Next, to assess changes in transcription as cells progressed along the trajectory, we modelled the transcription of each gene as a function of pseudotime [45]. We identified 1072 differentially transcribed genes (False discovery rate (FDR) < 0.01; S6 Table). Heatmap visualisation of the data revealed three common patterns of how transcription changed over pseudotime, including a gradual decrease, transient increase, and transient decrease (Fig 4C). Our assessment of *P. vivax* sporozoites over pseudotime provides further insights into gene usage, taking into account that cell transitions in these forms may occur more continuously.

P. vivax sporozoites have distinct transcriptomic signatures relative to *P. falciparum* sporozoites

Whereas *P. vivax* sporozoites can form hypnozoites in hepatocytes, *P. falciparum* sporozoites cannot. Therefore, we compared sporozoite transcriptomes from these two species to assess the extent to which their gene expression profiles differ and search for signatures of potential importance for hypnozoite formation. We first identified orthologous gene groupings to allow for the cross-species comparison [46]. After subsampling the *P. vivax* data to balance the proportions of *P. falciparum* [23] and *P. vivax* cells, we then combined the two species' datasets to produce an integrated UMAP (Fig 5A top). The projection revealed that sporozoites in the *P. vivax* and *P. falciparum* data mapped close together (Fig 5A top). The projection also revealed distinct populations of *P. vivax* and *P. falciparum* cells, with the latter grouped by anatomical location in the mosquito (Fig 5A bottom). The annotated *P. falciparum* data by mosquito location

and activation status allowed us to infer the transcriptomic status of *P. vivax* sporozoites. Using these annotations as a guide, we grouped the sporozoites into four clusters (Fig 5B). Cluster 1 (ORTHO_C1) represented midgut/recently invaded sporozoites; cluster 2 (ORTHO_C2), salivary gland sporozoites; and clusters 3 and 4 (ORTHO_3 and ORTHO_4), activating or activated sporozoites. More than half of the *P. vivax* sporozoite data (63%, 949/1500) clustered with the *P. falciparum* salivary gland sporozoites in ORTHO_C2 (Fig 5C and Fig S5A). Interestingly, some *P. vivax* sporozoites were assigned to clusters ORTHO_C1, ORTHO_C3 and ORTHO_C4, shedding light on their intrinsic heterogeneity within the salivary glands of the mosquito (Fig 5C and Fig S5A).

For each cluster, we identified genes that display greater transcription relative to the other clusters and are conserved in both species, which we denote as conserved markers. In total, we identified 12 conserved markers (adjusted p-value < 0.05; Fig S5C; S8 Table). Relative to the other clusters, only sporozoites in the midgut/recently invaded and salivary gland clusters (ORTHO_C1 and ORTHO_C2, respectively) had conserved gene signatures. We reasoned that the disproportionate number of sporozoites for each species in ORTHO_C3 and ORTHO_C4 (Fig 5C and Fig S5A) contributed to the lack of conserved markers detected. Of the genes with known function identified in ORTHO_C1, notable examples included sporozoite invasion-associated protein 1 (PVP01_0307900 | PF3D7_0408600), important for sporozoite exit from the mosquito midgut and colonization of the salivary gland [47] and sporozoite-specific protein S10 (PVP01_0304200 | PF3D7_0404800), a marker implicated in salivary gland invasion [22,25] (Fig S5C; S6 Table). In ORTHO_C2, genes linked to invasion (PVP01_1132600 | PF3D7_0616500::TRAP-like protein) and liver stage development (PVP01_0938800 | PF3D7_1137800::SPELD) were among the conserved markers identified (Fig S5C; S8 Table).

To elucidate species-specific transcription patterns within each cluster, we compared transcript levels between species. Each cluster contained orthologues transcribed solely in *P. vivax* or *P. falciparum* (Fig 5D left and Fig S5B; S9 Table). ORTHO_C4, which was composed almost entirely of *P. vivax* sporozoites, was excluded from the analysis. We thus focused our analyses on the genes detected in both species in clusters one through three (Fig 5D right and Fig S5B; S9 Table). In total, we identified 155 differentially transcribed genes (adjusted p-value < 0.05), 44 (28%) of which had unknown function (S10 Table). In Fig S6A, we highlight the top differentially transcribed genes within each cluster. At a broad level, genes associated with ion transport (PVP01_0317600 | PF3D7_0721900, PVP01_1014700 | PF3D7_0519200, PVP01_1117400 | PF3D7_1354400, PVP01_1242100 | PF3D7_1464700) displayed greater

transcription in ORTHO_C1 *P. vivax* sporozoites (adjusted p-value < 0.05, S10 Table). Alternatively, clusters ORTHO_C2 and ORTHO_C3, various genes associated with locomotion and motility (PVP01_1132600 | PF3D7_0616500, PVP01_1435400 | PF3D7_1216600, PVP01_1218700 | PF3D7_1335900) displayed greater transcription in *P. falciparum* (adjusted p-value < 0.05, S10 Table). Combined with the species-specific genes identified in each cluster, these differences highlight the distinct transcriptomic signatures between *P. vivax* and *P. falciparum*.

ORTHO_C3 and ORTHO_C4 contained sporozoites with transcriptomic signatures similar to *P. falciparum* activated sporozoites. As ORTHO_C4 was made up of primarily *P. vivax* cells, we sought to assess the extent to which sporozoites in this cluster differed from those in ORTHO_C3 containing both *P. falciparum* and *P. vivax* sporozoites. In total, we identified 98 differentially transcribed genes (adjusted p-value < 0.05, S11 Table), several (38/98; 38%) of which have no known function. Interestingly, genes that displayed significantly greater transcription in ORTHO_C4 (Fig 5E) contained Pfam domains associated with translational machinery (PF01200 and PF01253) and RNA-binding proteins (PF00076, PF00806), including mRNA-binding protein PUF2 (PVP01_0526500). PUF2 is an important eukaryotic cell-fate regulator [48], and plays a key-role in translationally repressing transcripts, including *uis3* and *uis4*, required during liver-stage development [49,50]. Interestingly, in the absence of PUF2 (Pfam: PF00806::PUF RNA binding repeat), salivary gland sporozoites may initiate exoerythrocytic development independently of the transmission-associated environmental cues [49]. These findings indicate although cellular morphology and invasion strategies are similar in sporozoites from the two species, their respective cellular states, as defined by their transcriptomic signatures, can vary drastically. Whether these differences in transcription play a role in determining the sporozoite's developmental fate upon reaching the liver warrants further investigation.

Comparative analyses of *P. vivax* sporozoite and blood-stage transcriptomes reveals conserved and stage-specific signatures

To assess gene usage across different stages of the parasite's life cycle, we compared our sporozoite data with publicly-available scRNA-seq data from *P. vivax* blood stages [26]. Before data integration, we realigned the blood-stage sequencing data to account for the UTR additions to the *P. vivax* gene models. As expected, we found a 1.4-fold gain in the number of reads mapping to gene loci when the UTRs were included in the alignment (Fig S7A). Using the cell and gene filtering pipeline established for the sporozoite scRNA-seq data, 12,469 blood-

stages parasites were assessed (Fig S7B). We detected a median of 682 genes and 1,442 UMIs per cell (Fig S7C). Furthermore, low-dimensional representation of the data confirmed distinct transcriptomic signatures across each sample (Fig S7D).

After cell and gene filtering, we integrated the *P. vivax* blood-stage data with our sporozoite data (Fig 6A, Fig S7E) and performed differential transcription analysis. We identified 208 differentially transcribed genes, 36 of which remain uncharacterised (minimum expression in 50% of cells, adjusted p-value < 0.1; S13 Table). Among the differentially transcribed genes, 59 displayed sporozoite-specific transcription (adjusted p-value < 0.01; S13 Table). As highlighted in Fig 6B, sporozoite-specific markers encoded for transcripts involved in sporozoite development, maturation, and host-cell infection [35–38,51,52]. Furthermore, we identified a small proportion of transcripts (18/208, 8%) present in a similar percentage of sporozoite and erythrocyte forms but with significantly higher transcriptional abundance in sporozoites (Fig 6C; S13 Table). Together, this integrated analysis reveals various stage-specific markers and provides the framework for creating a comprehensive reference map for *P. vivax.*

Discussion

Our scRNA-seq data reveals transcriptional differences among *P. vivax* sporozoites at a resolution previously unattainable with bulk transcriptome-wide approaches. As is expected with droplet-based single-cell capture technologies, a comparison of our scRNA-seq data with that of bulk methods reveals a reduced overall number of genes detected using the single-cell approach [19,20,53]. However, we find that the detection of highly transcribed sporozoite genes is achieved across technologies. Herein we also describe improved approaches for processing and analysing scRNA-seq data useful for the wider community. First, our overall gene detection rate was dramatically improved by including each genes' UTRs in our alignment strategy. Second, clustering and pseudotime analyses were useful for providing new insights into transcription patterns amongst *P. vivax* sporozoites in the salivary glands of mosquitos.

Visualising the data in low-dimensional space reveals transcriptional heterogeneity amongst sporozoites. After assigning the sporozoites to clusters and performing differential transcriptional analysis, we identified annotated and unannotated genes underlying this heterogeneity. We found that some sporozoites isolated from the salivary glands show increased transcriptional activity of orthologous genes previously shown to be transcribed in *P. berghei* midgut sporozoites [22,25]. We interpret the detection of these markers in *P. vivax* to

represent sporozoites that have recently invaded the salivary glands. However, we also found sporozoites that display lower gene usage, which we hypothesize is indicative of maturation. The pseudotime analysis serves to complement the cluster-based assessment of sporozoites. The ordering of sporozoites reveals a gradual decrease in the transcription of most genes. These findings likely recapitulate the transcription changes occurring as the sporozoite transitions from a recently invaded salivary gland sporozoite to a mature one. Translational repression of mRNA is one molecular mechanism sporozoites use to ensure timely production of proteins upon entering its host (reviewed in [54,55]). Our observation of the low number of UMIs and genes detected per cell, which corresponds with other assessments of *Plasmodium* sporozoites at single-cell resolution [22–25], suggests that transcriptional repression may also play a role in the sporozoites' preparation for host invasion.

It has been hypothesized that *P. vivax* sporozoites are pre-programmed to become replicating schizonts or hypnozoites [16,17]. However, evidence to support this hypothesis at the molecular level is lacking. We proposed that the transcriptomic analysis of individual sporozoites could test this hypothesis and might support identification of two distinct sporozoite populations, with one destined for normal hepatic development and the other for hypnozoite formation. By comparison, we expected sporozoites from *P. falciparum*, which do not form hypnozoites, would have the first but lack the second population. Our comparative analyses identify a large population of *P. vivax* and *P. falciparum* sporozoites that have conserved patterns in gene usage for various markers implicated in sporozoite biology. These include the genes encoding for sporozoite invasion-associated protein 1, TRAP-like protein, and sporozoite surface protein essential for liver stage development [47,52,56]. Interestingly, distinguishing *P. vivax* from *P. falciparum* were a subset (~25%) of *P. vivax* sporozoites transcribing genes (e.g. *puf2*) associated with translational regulation and repression. Noting the role translational repression plays through RNA-binding proteins, including PUF2, in developmental fating in eukaryotes [57], it is intriguing to speculate on possible links between this population and the bradysporozoites [16] proposed to be destined for hypnozoite formation.

Future analyses comparing *P. vivax* to the other sporozoites derived from parasites that cause relapsing malaria, such as *P. cynomolgi* and *P. ovale,* may help shed light on the relapsing-specific factors associated with the parasite's developmental fate. However, the differences in methodology used to generate sporozoite scRNA-seq datasets should be considered for these future studies. Of note, scRNA-seq of *P. falciparum* sporozoites was performed after dissecting salivary glands into Schneider's insect medium and then sequencing

individual sporozoites following Accudenz purification, immunofluorescent staining and fluorescence-activated cell sorting [23]. In this study we used only a density gradient to purify sporozoites from salivary gland debris before loading them onto a 10x Genomics' controller to generate single-cell RNA libraries. Both collection, purification, and capture workflows involve 1-3 hr of time from dissection to RNA capture, during which time the buffers and temperature used could impact sporozoite activation state [21,58]. Additionally, for both the *P. vivax* and *P. falciparum* datasets, pseudotime analysis was found useful for understanding the developmental state of sporozoites, meaning the day, and possibly hour, of dissection post blood-meal could impact comparisons. Despite these differences, we note the substantial overlap in *P. vivax* and *P. falciparum* developmental states in low dimensional space. Still, for future studies these parameters should be standardised as much as possible to remove potential technical artefacts from comparisons.

Previous bulk sequencing analyses comparing *P. vivax* sporozoites and blood-stage parasites have revealed distinct transcription patterns between stages [19,59,60]. Here, we corroborate these findings and show that this variation is detectable using scRNA-seq technology. Our comparison of *P.* vivax sporozoites and blood stages highlights differences in transcription that may provide insights into the factors that allow the parasite to persist in different environments. Furthermore, our integrated analysis sets the stage for future additions, with the objective of generating a comprehensive *P. vivax* single-cell atlas. Our data serves as a new resource for the malaria research community, providing a detailed assessment of *P. vivax* gene usage in sporozoites at single-cell resolution. We anticipate that the dataset will serve as a launchpad to address other gaps in knowledge linked to *P. vivax* biology, including the extent to which various strains differ at the transcriptome level and how gene usage in individual sporozoites compares to other stages in the parasite's life cycle.

Materials and methods

Ethics statement

Research procedures and protocols for obtaining blood from patients were reviewed and approved by the Cambodian National Ethics Committee for Health Research (approval number: #113NECHR). Protocols conformed to the World Medical Association Helsinki Declaration on Ethical Principles for Medical Research Involving Human Subjects (version 2002). Informed written consent was obtained from all volunteers or legal guardians.

Blood samples, mosquitos, and infections

Blood samples from symptomatic patients infected with *P. vivax* were collected at local health facilities in Mondulkiri province (Kaev Seima) in Eastern Cambodia from September to November 2019 (Fig 1A). Following a *P. vivax* gametocyte-containing blood meal, *Anopheles dirus* mosquitoes were maintained at 26°C on a 12hr:12hr light:dark cycle and fed with 10% sucrose + 0.05% PABA solution. *An. dirus* found positive for *P. vivax* oocysts at six days post-feeding were transported to the Institut Pasteur of Cambodia Insectary Facility in Phnom Penh, Cambodia, where they were maintained under the same conditions described above.

Sporozoite isolations

P. vivax sporozoites were isolated from the salivary glands of female *An. dirus* mosquitoes 16-18 days after an infectious blood-meal. A team of technicians performed dissections for a maximum period of one hour. Generally, ~75 to 100 mosquitoes were dissected in each one-hour sitting. Dissections were performed under a stereomicroscope, and salivary glands were placed in a microcentrifuge tube containing ice-cold Hanks Balanced Salt Solution. Sporozoites were then released from the salivary glands via manual disruption using a microcentrifuge pestle, and immediately purified using a discontinuous density gradient protocol adapted from Kennedy and colleagues [61], and as described previously [25]. After purification, sporozoite mixtures were diluted in HBSS to 1000 sporozoites/mL and were held on ice until further processing.

Hepatocyte infections and liver-stage assessment

P. vivax hepatocyte infections were performed as previously described [27]. Briefly, primary human hepatocytes (BioIVT) were seeded 2-3 days prior to infection with 15,000-20,000 sporozoites per well. Media was exchanged with fresh CP media (BioIVT) containing antibiotics the day after infection and every 2-3 days thereafter. At 12 days post-infection, cultures were fixed with 4% paraformaldehyde in 1X PBS. Fixed cultures were stained overnight at 4ºC with recombinant mouse anti-*P. vivax* UIS4 antibody diluted 1:25,000 in a stain buffer (0.03% TritonX-100 and 1% (w/v) BSA in 1X PBS) [62]. Cultures were then washed thrice with 1X PBS and then stained overnight at 4ºC with 1:1000 rabbit anti-mouse AlexaFluor ™488-conjugated antibody diluted 1:1,000 in stain buffer. Cultures were then washed thrice with 1X PBS and counterstained with 1 mg/mL Hoechst 33342 to detect parasite and host cell nuclear

DNA. Infected hepatocytes were imaged on a Lionheart (Biotek). Quantification of nuclei and liver forms was performed using Gen5 high content analysis software (Biotek).

Single-cell partitioning, library preparation, and sequencing

Approximately 5000-8000 sporozoites were loaded on a version 3- specific Chromium Chip. Chips containing sporozoite suspensions, Gel Beads in Emulsion (GEMs), and reverse transcription reagents were placed in a Chromium controller for single-cell partitioning and cellular barcoding. Barcoded cDNA libraries were generated according to the v3 Chromium Single Cell 3' gene expression protocol. cDNA libraries were loaded on individual flow cell lanes and sequenced using a HiSeq X Ten platform (Illumina) at Macrogen (Seoul, Korea). See Table S1 for sequencing statistics.

Read alignment, cellular barcode assignment, and quantification

The *P. vivax* P01 genome (version 3, October 2020) and its corresponding general feature format (gff) file (which contained UTR coordinates) were downloaded from ftp://ftp.sanger.ac.uk/pub/genedb/releases/latest/PvivaxP01 and used to create a genome index with STAR (v2.7.3a) [63,64] with options: --runMode genomeGenerate –genomeSAindexNbases 11 –sjdbOverhang 90 (for v3 libraries), or 74 (for libraries derived from [26]). Mapping, demultiplexing and gene quantification was performed with STARsolo and the following options specified: --soloType CB_UMI_Simple --soloCBlen 16 --soloUMIlen 12 (for v3 libraries) or 10 (for v2 libraries) --soloCBwhitelist /path/to/10x/version/specific/whitelist --alignIntronMin 1 --alignIntronMax 2756 --soloUMIfiltering MultiGeneUMI --soloFeatures Gene.

The *An. dirus* WRAIR2 genome and its corresponding GFF were downloaded from VectorBase (v49). We generated a genome index in STAR (v2.7.3a) with the same parameters as for the *P. vivax* P01 genome.

For a schematic of the alignment strategies, refer to Fig S2A and Fig S2C. A summary of read alignment statistics for each sample with- and without- the UTR information are found in S2 and S12 Tables for the sporozoite and blood-stage data, respectively.

Filtering and normalisation of scRNA-seq count matrices

The unfiltered (raw) matrix, features, and barcodes files generated from STAR were imported into R (version 4) [65]. We first removed rRNA encoding genes and then used

emptyDrops function to distinguish between droplets containing cells and those only with ambient RNA to be discarded [66] with a lower library size limit of 40 to account for the low mRNA amounts in sporozoites (FDR < 0.001). We next removed cells with less than 60 unique genes detected in each cell library. Last, we filtered out lowly detected genes, retaining genes detected in at least two cells with more than two unique molecular identifier (UMI) counts. Post-cell and gene filtering, the data from each replicate were normalised using Seurat's 'LogNormalize' function with the default parameters selected [43].

Integration of *P. vivax* sporozoite scRNA-seq data

Filtered, normalised matrices from the three replicates were merged in a manner described in the Seurat (version 4) vignette, *Introduction to scRNA-seq integration*, available on the Satija Lab's website (https://satijalab.org/seurat/articles/integration_introduction.html). Briefly, highly variable features were identified in each replicate using the 'FindVariableFeatures' function with the following parameters selected: selection method = "vst", features = (genes # of detected in dataset) * 0.3. Next, integration anchors were identified using the 'FindIntegrationAnchors' function with its default settings. Lastly, using these anchors, the three datasets were integrated using the 'IntegrateData' function with its default settings.

Dimension reduction, clustering, and cluster marker identification

Post integration, data were scaled, and dimension reduction was performed using principal component analysis (PCA) to visualise the data in low-dimensional space. Next, the UMAP dimension reduction was performed using the RunUMAP function with the parameters dims = 1:30 and umap.method = "umap-learn", n.neighbours = 20 and min.dist = 0.5. Next, an unsupervised graph-based clustering approach was used to predict cell communities. First, k-nearest neighbours were identified, and a shared nearest neighbour graph was constructed using the FindNeighbours function under the default settings. Cell communities (clusters) were then identified using the FindClusters function with the Leiden algorithm (algorithm = 4) selected. Clustering was performed at various resolutions (resolution = 0.1:1), and cluster stabilities were assessed using a Clustering tree plot [67].

To detect cluster-specific markers (differentially transcribed genes), the Seurat function FinalAllMarkers was used with the following parameters: test.use = "wilcox", min.pct = 0.3, min.diff.pct = 0.1, only.pos = TRUE, assay = "originalexp". Differentially transcribed genes were considered significant if the adjusted p value was below 0.05.

Trajectory analysis

To infer the developmental trajectory of sporozoites, we used the Slingshot package [44] to uncover the global structure of clusters of cells and convert this structure into a smoothed lineage representing "pseudotime". Lineages were first generated using the getLineages function on the UMAP embeddings generated previously (described in *Dimension reduction, clustering and cluster marker identification*). Cluster C1 was selected as the starting cluster because it contained putative immature salivary gland sporozoite markers. Next, smoothed lineage curves were constructed using the getCurves function with the default parameters. We then used the tradeSeq package [45] to analyse transcription along the trajectory. To this end, we ran the fitGAM function on the SlingshotDataset to fit a negative binomial general additive model on the data. Based on the fitted models, the AssociationTest with default settings selected was used to test transcription changes across pseudotime. Genes were considered significantly associated with a change over pseudotime at a false discovery rate below 0.01.

Inter-species comparison of *Plasmodium* spp. sporozoites

We grouped orthologous genes of *P. vivax P01, P. cynomolgi M, P. cynomolgi B, P. berghei ANKA, P. falciparum 3D7, P. yoelli yoelli 17X, P. chabaudi chabaudi, P. knowlesi H, P. malariae UG01, P. ovale curtisi GH01 and Toxoplasma gondii ME49* using OrthoFinder [46]. Parameters were kept at default and gene fasta files for input were obtained from either *PlasmoDB* or *ToxoDB*, release version 51. From the 'Orthogroups' analysis, the tab-delimited output file was used to extract the species-specific gene IDs for *P. vivax* and *P. falciparum* and match these with a universal orthogroup ID (S7 Table). We used this orthogroup ID to replace rownames corresponding to *P. vivax* or *P. falciparum* gene IDs in the count matrices of *P. vivax* sporozoites (this study) and *P. falciparum* sporozoites [23]. Orthogroup IDs for merge were only retained if they had only one entry per species. In a *P. vivax*-centric approach, we retained orthogroup IDs if either *P. falciparum, P. berghei* ANKA, *P. cynomolgi M* or *P. cynomolgi* B had at least one corresponding gene orthologue. *P. vivax* data were processed as described earlier (section *Filtering and normalization of scRNA-seq count matrices*) and *P. falciparum* data was obtained from https://github.com/vhowick/pf_moz_stage_atlas/tree/master/counts_and_metadata. Following the replacement of gene IDs with new orthogroup IDs, we additionally performed another round of cell filtering to account for changes in gene count per cell information with the removal of some genes without equivalent orthogroup IDs. Data integration were also performed as

described earlier (section *Integration of P. vivax sporozoite scRNA-seq datasets*). The *P. vivax* data were randomly subset (500 sporozoites per replicate) for subsequent analyses to match the proportion of cells in the *P. falciparum* dataset prior to integration and account for the disproportionate number of sporozoites between the two species. Clusters of sporozoites were identified using ‘FindNeighbours’ function using the ‘pca’ reduction, dims = 1:15, k.param = 20, and ‘FindClusters’ function with the Leiden clustering algorithm (algorithm = 4) at a resolution of 0.2. Marker genes for the defined clusters were identified with either the 'FinderConservedMarkers' or 'FindMarkers' functions (Seurat) using a Wilcoxon Rank Sum test. The number of cells, percent difference, and fold-change parameters for each of the analyses are indicated in the R markdown document provided.

Integration of *P. vivax* sporozoite and blood stage scRNA-seq data

Filtering and normalisation for the *P. vivax* blood-stage scRNA-seq data was performed in the same manner used for the sporozoite scRNA-seq data described in *Filtering and normalization of scRNA-seq count matrices*. Of the 10 *P. vivax* blood-stage replicates, we used the 7 generated from samples without chloroquine treatment. Before integration, variable features for the combined sporozoite (3) and combined blood-stage (7) datasets were identified using the FindVariableFeatures function with the following parameters: selection method = "vst", features = (genes # of detected in dataset) * 0.3. Next, we used the FindIntegrationAnchors function to identify anchors between the sporozoite and blood-stage datasets. Guided by the recommendations provided by the Satija lab's vignette *Fast integration using reciprocal PCA (RPCA)* (https://satijalab.org/seurat/articles/integration_rpca.html), namely when cells in one dataset have no matching type in the other, we selected the RPCA parameter in FindIntegrationAnchors (reduction = "rpca") to identify anchors between the sporozoite and blood-stage data. Last, using these anchors, the datasets were integrated using the IntegrateData function with its default settings. Assessment of differentially transcribed genes in sporozoite and blood-stage parasites were performed in the same manner described in *Differential genes transcription analysis* with additional parameters indicated in the provided R markdown files.

Contact for reagent and resource sharing

Further information and requests for resources and reagents should be directed to the Lead Contact, Ivo Mueller (e-mail: mueller@wehi.edu.au).

Data Availability:

All raw sequencing data generated from *P. vivax* sporozoites in this study will be deposited and accessible in the European Nucleotide Archive (www.ebi.ac.uk/ena/) upon acceptance of the manuscript.

P. vivax blood-stage scRNA-seq data were downloaded from NCBI's Short Read Archive (Bioproject ID: PRJNA603327).

P. falciparum sporozoite scRNA-seq data were obtained from: https://github.com/vhowick/pf_moz_stage_atlas

Scripts and supporting files will be available on GitHub upon acceptance of the manuscript.

Archived scripts and output files as at time of publication will be available on Zenodo.com upon acceptance of the manuscript.

Acknowledgments

We thank the *P. vivax* patients of Mondulkiri Province, Cambodia, for participating in this study. We thank the Institut Pasteur insectary staff (Makara Pring, Koeun Kaing, Nora Sambath) for *An. dirus* mosquito colony maintenance, the laboratory staff (Eakpor Piv, Chansophea Chhin, Sreyvouch Phen, Chansovandan Chhun, Sivcheng Phal, Baura Tat) for assistance with the mosquito dissections and the *in vitro* assays, and the field site manager (Saorin Kim) for logistical assistance.

References

1. World Health Organization. World malaria report 2020. WHO. 2020. Available: https://www.who.int/publications/i/item/9789240015791

2. Battle KE, Baird KJ. The global burden of Plasmodium vivax malaria is obscure and insidious. PLOS Medicine. 2021;18: e1003799. doi:10.1371/JOURNAL.PMED.1003799

3. Battle KE, Lucas TCD, Nguyen M, Howes RE, Nandi AK, Twohig KA, et al. Mapping the global endemicity and clinical burden of Plasmodium vivax, 2000–17: a spatial and temporal modelling study. The Lancet. 2019;394: 332–343. doi:10.1016/S0140-6736(19)31096-7

4. Weiss DJ, Lucas TCD, Nguyen M, Nandi AK, Bisanzio D, Battle KE, et al. Mapping the global prevalence, incidence, and mortality of Plasmodium falciparum, 2000–17: a spatial and temporal modelling study. The Lancet. 2019;394: 322–331. doi:10.1016/S0140-

6736(19)31097-9/ATTACHMENT/33EB90A6-D959-4261-8281-5E12C6273433/MMC1.PDF

5. Amino R, Thiberge S, Martin B, Celli S, Shorte S, Frischknecht F, et al. Quantitative imaging of Plasmodium transmission from mosquito to mammal. Nature Medicine. 2006;12: 220–224. doi:10.1038/nm1350

6. Krotoski WA. Discovery of the hypnozoite and a new theory of malarial relapse. Transactions of the Royal Society of Tropical Medicine and Hygiene. 1985;79: 1. doi:10.1016/0035-9203(85)90221-4/2/79-1-1.PDF.GIF

7. Krotoski WA, Collins WE, Bray RS, Garnham PC, Cogswell FB, Gwadz RW, et al. Demonstration of hypnozoites in sporozoite-transmitted Plasmodium vivax infection. The American journal of tropical medicine and hygiene. 1982;31: 1291–1293. doi:10.4269/AJTMH.1982.31.1291

8. Robinson LJ, Wampfler R, Betuela I, Karl S, White MT, Li Wai Suen CSN, et al. Strategies for Understanding and Reducing the Plasmodium vivax and Plasmodium ovale Hypnozoite Reservoir in Papua New Guinean Children: A Randomised Placebo-Controlled Trial and Mathematical Model. PLOS Medicine. 2015;12: e1001891. doi:10.1371/journal.pmed.1001891

9. Adekunle AI, Pinkevych M, McGready R, Luxemburger C, White LJ, Nosten F, et al. Modeling the Dynamics of Plasmodium vivax Infection and Hypnozoite Reactivation In Vivo. PLOS Neglected Tropical Diseases. 2015;9: e0003595. doi:10.1371/journal.pntd.0003595

10. Commons RJ, Simpson JA, Watson J, White NJ, Price RN. Estimating the Proportion of Plasmodium vivax Recurrences Caused by Relapse: A Systematic Review and Meta-Analysis. American Journal of Tropical Medicine and Hygiene. 2020;103: 1094–1099. doi:10.4269/ajtmh.20-0186

11. Sattabongkot J, Suansomjit C, Nguitragool W, Sirichaisinthop J, Warit S, Tiensuwan M, et al. Prevalence of asymptomatic Plasmodium infections with sub-microscopic parasite densities in the northwestern border of Thailand: A potential threat to malaria elimination. Malaria Journal. 2018;17. doi:10.1186/s12936-018-2476-1

12. White MT, Walker P, Karl S, Hetzel MW, Freeman T, Waltmann A, et al. Mathematical modelling of the impact of expanding levels of malaria control interventions on Plasmodium vivax. Nature Communications 2018 9:1. 2018;9: 1–10. doi:10.1038/s41467-018-05860-8

13. Huldén L, Huldén L, Heliövaara K. Natural relapses in vivax malaria induced by Anopheles mosquitoes. Malaria journal. 2008;7. doi:10.1186/1475-2875-7-64

14. Battle KE, Karhunen MS, Bhatt S, Gething PW, Howes RE, Golding N, et al. Geographical variation in Plasmodium vivax relapse. Malaria Journal. 2014;13. doi:10.1186/1475-2875-13-144

15. White NJ. Determinants of relapse periodicity in Plasmodium vivax malaria. Malaria Journal. 2011. doi:10.1186/1475-2875-10-297

16. Lysenko AJ, Beljaev AE, Rybalka VM. Population studies of Plasmodium vivax. 1. The theory of polymorphism of sporozoites and epidemiological phenomena of tertian malaria. Bulletin of the World Health Organization. 1977.

17. Ungureanu E, Killick-Kendrick R, Garnham PCC, Branzei P, Romanescu C, Shute PG. Prepatent periods of a tropical strain of Plasmodium vivax after inoculations of tenfold dilutions of sporozoites. Transactions of the Royal Society of Tropical Medicine and Hygiene. 1976;70: 482–483. doi:10.1016/0035-9203(76)90133-4

18. Mikolajczak SA, Vaughan AM, Kangwanrangsan N, Roobsoong W, Fishbaugher M, Yimamnuaychok N, et al. Plasmodium vivax liver stage development and hypnozoite persistence in human liver-chimeric mice. Cell Host and Microbe. 2015;17: 526–535. doi:10.1016/j.chom.2015.02.011

19. Muller I, Jex AR, Kappe SHI, Mikolajczak SA, Sattabongkot J, Patrapuvich R, et al. Transcriptome and histone epigenome of Plasmodium vivax salivary-gland sporozoites point to tight regulatory control and mechanisms for liver-stage differentiation in relapsing malaria. International Journal for Parasitology. 2019;49: 501–513. doi:10.1016/J.IJPARA.2019.02.007

20. Westenberger SJ, McClean CM, Chattopadhyay R, Dharia N v., Carlton JM, Barnwell JW, et al. A Systems-Based Analysis of Plasmodium vivax Lifecycle Transcription from Human to Mosquito. PLoS Neglected Tropical Diseases. 2010;4: e653. doi:10.1371/journal.pntd.0000653

21. Roth A, Adapa SR, Zhang M, Liao X, Saxena V, Goffe R, et al. Unraveling the Plasmodium vivax sporozoite transcriptional journey from mosquito vector to human host. Scientific Reports. 2018;8: 12183. doi:10.1038/s41598-018-30713-1

22. Bogale HN, Pascini T v., Kanatani S, Sá JM, Wellems TE, Sinnis P, et al. Transcriptional heterogeneity and tightly regulated changes in gene expression during Plasmodium berghei sporozoite development. Proceedings of the National Academy of Sciences of the United States of America. 2021;118. doi:10.1073/pnas.2023438118

23. Real E, Howick VM, Dahalan FA, Witmer K, Cudini J, Andradi-Brown C, et al. A single-cell atlas of Plasmodium falciparum transmission through the mosquito. Nature Communications. 2021;12: 3196. doi:10.1038/s41467-021-23434-z

24. Howick VM, Russell AJC, Andrews T, Heaton H, Reid AJ, Natarajan K, et al. The Malaria Cell Atlas: Single parasite transcriptomes across the complete Plasmodium life cycle. Science. 2019;365: eaaw2619. doi:10.1126/SCIENCE.AAW2619

25. Ruberto AA, Bourke C, Merienne N, Obadia T, Amino R, Mueller I. Single-cell RNA sequencing reveals developmental heterogeneity among Plasmodium berghei sporozoites. Scientific Reports. 2021;11: 4127. doi:10.1038/s41598-021-82914-w

26. Sà JM, Cannon M v., Caleon RL, Wellems TE, Serre D. Single-cell transcription analysis of Plasmodium vivax blood-stage parasites identifies stage- And species-specific profiles of expression. PLoS Biology. 2020;18: e3000711. doi:10.1371/journal.pbio.3000711

27. Roth A, Maher SP, Conway AJ, Ubalee R, Chaumeau V, Andolina C, et al. A comprehensive model for assessment of liver stage therapies targeting Plasmodium

vivax and Plasmodium falciparum. Nature Communications. 2018;9: 1837. doi:10.1038/s41467-018-04221-9

28. Klein AM, Mazutis L, Akartuna I, Tallapragada N, Veres A, Li V, et al. Droplet barcoding for single-cell transcriptomics applied to embryonic stem cells. Cell. 2015;161: 1187–1201. doi:10.1016/j.cell.2015.04.044

29. Macosko EZ, Basu A, Satija R, Nemesh J, Shekhar K, Goldman M, et al. Highly parallel genome-wide expression profiling of individual cells using nanoliter droplets. Cell. 2015;161: 1202–1214. doi:10.1016/j.cell.2015.05.002

30. Zheng GXY, Terry JM, Belgrader P, Ryvkin P, Bent ZW, Wilson R, et al. Massively parallel digital transcriptional profiling of single cells. Nature Communications. 2017;8. doi:10.1038/ncomms14049

31. Shields EJ, Sorida M, Sheng L, Sieriebriennikov B, Ding L, Bonasio R. Genome annotation with long RNA reads reveals new patterns of gene expression in an ant brain. bioRxiv. 2021; 2021.04.20.440671. doi:10.1101/2021.04.20.440671

32. Packer JS, Zhu Q, Huynh C, Sivaramakrishnan P, Preston E, Dueck H, et al. A lineage-resolved molecular atlas of C. Elegans embryogenesis at single-cell resolution. Science. 2019;365. doi:10.1126/science.aax1971

33. Siegel S v., Chappell L, Hostetler JB, Amaratunga C, Suon S, Böhme U, et al. Analysis of Plasmodium vivax schizont transcriptomes from field isolates reveals heterogeneity of expression of genes involved in host-parasite interactions. Scientific Reports. 2020;10: 16667. doi:10.1038/s41598-020-73562-7

34. Kivioja T, Vähärautio A, Karlsson K, Bonke M, Enge M, Linnarsson S, et al. Counting absolute numbers of molecules using unique molecular identifiers. Nature methods. 2011;9: 72–74. doi:10.1038/NMETH.1778

35. Sinnis P, Clavijo P, Fenyö D, Chait BT, Cerami C, Nussenzweig V. Structural and functional properties of region II-plus of the malaria circumsporozoite protein. Journal of Experimental Medicine. 1994;180. doi:10.1084/jem.180.1.297

36. Coppi A, Natarajan R, Pradel G, Bennett BL, James ER, Roggero MA, et al. The malaria circumsporozoite protein has two functional domains, each with distinct roles as sporozoites journey from mosquito to mammalian host. Journal of Experimental Medicine. 2011;208. doi:10.1084/jem.20101488

37. Ishino T, Yano K, Chinzei Y, Yuda M. Cell-passage activity is required for the malarial parasite to cross the liver sinusoidal cell layer. PLoS Biology. 2004;2. doi:10.1371/journal.pbio.0020004

38. Talman AM, Lacroix C, Marques SR, Blagborough AM, Carzaniga R, Ménard R, et al. PbGEST mediates malaria transmission to both mosquito and vertebrate host. Molecular Microbiology. 2011;82. doi:10.1111/j.1365-2958.2011.07823.x

39. Mueller AK, Camargo N, Kaiser K, Andorfer C, Frevert U, Matuschewski K, et al. Plasmodium liver stage developmental arrest by depletion of a protein at the parasite–

host interface. Proceedings of the National Academy of Sciences. 2005;102: 3022–3027. doi:10.1073/PNAS.0408442102

40. Matuschewski K, Ross J, Brown SM, Kaiser K, Nussenzweig V, Kappe SHI. Infectivity-associated Changes in the Transcriptional Repertoire of the Malaria Parasite Sporozoite Stage *. Journal of Biological Chemistry. 2002;277: 41948–41953. doi:10.1074/JBC.M207315200

41. Real E, Rodrigues L, Cabal GG, Enguita FJ, Mancio-Silva L, Mello-Vieira J, et al. Plasmodium UIS3 sequesters host LC3 to avoid elimination by autophagy in hepatocytes. Nature Microbiology 2017 3:1. 2017;3: 17–25. doi:10.1038/s41564-017-0054-x

42. Traag VA, Waltman L, van Eck NJ. From Louvain to Leiden: guaranteeing well-connected communities. Scientific Reports. 2019;9: 1–12. doi:10.1038/s41598-019-41695-z

43. Stuart T, Butler A, Hoffman P, Hafemeister C, Papalexi E, Mauck WM, et al. Comprehensive Integration of Single-Cell Data. Cell. 2019;177: 1888-1902.e21. doi:10.1016/j.cell.2019.05.031

44. Street K, Risso D, Fletcher RB, Das D, Ngai J, Yosef N, et al. Slingshot: Cell lineage and pseudotime inference for single-cell transcriptomics. BMC Genomics. 2018;19. doi:10.1186/s12864-018-4772-0

45. van den Berge K, Roux de Bézieux H, Street K, Saelens W, Cannoodt R, Saeys Y, et al. Trajectory-based differential expression analysis for single-cell sequencing data. Nature Communications. 2020;11. doi:10.1038/s41467-020-14766-3

46. Emms DM, Kelly S. OrthoFinder: Phylogenetic orthology inference for comparative genomics. Genome Biology. 2019;20: 238. doi:10.1186/s13059-019-1832-y

47. Engelmann S, Silvie O, Matuschewski K. Disruption of Plasmodium sporozoite transmission by depletion of sporozoite invasion-associated protein 1. Eukaryotic Cell. 2009;8. doi:10.1128/EC.00347-08

48. Zhang B, Gallegos M, Puoti A, Durkin E, Fields S, Kimble J, et al. A conserved RNA-binding protein that regulates sexual fates in the C. elegans hermaphrodite germ line. Nature 1997 390:6659. 1997;390: 477–484. doi:10.1038/37297

49. Gomes-Santos CSS, Braks J, Prudêncio M, Carret C, Gomes AR, Pain A, et al. Transition of Plasmodium sporozoites into liver stage-like forms is regulated by the RNA binding protein Pumilio. PLoS Pathogens. 2011;7. doi:10.1371/journal.ppat.1002046

50. Lindner SE, Mikolajczak SA, Vaughan AM, Moon W, Joyce BR, Sullivan WJ, et al. Perturbations of PlasmodiumPuf2 expression and RNA-seq of Puf2-deficient sporozoites reveal a critical role in maintaining RNA homeostasis and parasite transmissibility. Cellular Microbiology. 2013;15: 1266–1283. doi:10.1111/cmi.12116

51. Cerami C, Frevert U, Sinnis P, Takacs B, Clavijo P, Santos MJ, et al. The basolateral domain of the hepatocyte plasma membrane bears receptors for the circumsporozoite protein of plasmodium falciparum sporozoites. Cell. 1992;70. doi:10.1016/0092-8674(92)90251-7

52. Al-Nihmi FMA, Kolli SK, Reddy SR, Mastan BS, Togiri J, Maruthi M, et al. A Novel and Conserved Plasmodium Sporozoite Membrane Protein SPELD is Required for Maturation of Exo-erythrocytic Forms. Scientific Reports. 2017;7. doi:10.1038/srep40407

53. Haque A, Engel J, Teichmann SA, Lönnberg T. A practical guide to single-cell RNA-sequencing for biomedical research and clinical applications. Genome Medicine. BioMed Central Ltd.; 2017. pp. 1–12. doi:10.1186/s13073-017-0467-4

54. Schäfer C, Zanghi G, Vaughan AM, Kappe SHI. Plasmodium vivax Latent Liver Stage Infection and Relapse: Biological Insights and New Experimental Tools . Annual Review of Microbiology. 2021;75. doi:10.1146/annurev-micro-032421-061155

55. Briquet S, Marinach C, Silvie O, Vaquero C. Preparing for Transmission: Gene Regulation in Plasmodium Sporozoites. Frontiers in Cellular and Infection Microbiology. Frontiers Media S.A.; 2021. p. 907. doi:10.3389/fcimb.2020.618430

56. Heiss K, Nie H, Kumar S, Daly TM, Bergman LW, Matuschewski K, et al. Functional Characterization of a Redundant Plasmodium TRAP Family Invasin, TRAP-Like Protein, by Aldolase Binding and a Genetic Complementation Test Mailing address for. EUKARYOTIC CELL. 2008;7: 1062–1070. doi:10.1128/EC.00089-08

57. Kong J, Lasko P. Translational control in cellular and developmental processes. Nature Reviews Genetics 2012 13:6. 2012;13: 383–394. doi:10.1038/nrg3184

58. Hegge S, Kudryashev M, Barniol L, Frischknecht F. Key factors regulating Plasmodium berghei sporozoite survival and transformation revealed by an automated visual assay. FASEB journal : official publication of the Federation of American Societies for Experimental Biology. 2010;24: 5003–5012. doi:10.1096/FJ.10-164814

59. Bozdech Z, Mok S, Hu G, Imwong M, Jaidee A, Russell B, et al. The transcriptome of Plasmodium vivax reveals divergence and diversity of transcriptional regulation in malaria parasites. Proceedings of the National Academy of Sciences of the United States of America. 2008;105: 16290–16295. doi:10.1073/pnas.0807404105

60. Zhu L, Mok S, Imwong M, Jaidee A, Russell B, Nosten F, et al. New insights into the Plasmodium vivax transcriptome using RNA-Seq. Scientific Reports. 2016;6. doi:10.1038/srep20498

61. Kennedy M, Fishbaugher ME, Vaughan AM, Patrapuvich R, Boonhok R, Yimamnuaychok N, et al. A rapid and scalable density gradient purification method for Plasmodium sporozoites. Malaria Journal. 2012;11: 421. doi:10.1186/1475-2875-11-421

62. Schafer C, Dambrauskas N, Steel RW, Carbonetti S, Chuenchob V, Flannery EL, et al. A recombinant antibody against Plasmodium vivax UIS4 for distinguishing replicating from dormant liver stages. Malaria Journal. 2018;17: 370. doi:10.1186/s12936-018-2519-7

63. Kaminow B, Yunusov D, Dobin A. STARsolo: accurate, fast and versatile mapping/quantification of single-cell and single-nucleus RNA-seq data. bioRxiv. 2021; 2021.05.05.442755. doi:10.1101/2021.05.05.442755

64. Dobin A, Davis CA, Schlesinger F, Drenkow J, Zaleski C, Jha S, et al. STAR: Ultrafast universal RNA-seq aligner. Bioinformatics. 2013;29: 15–21. doi:10.1093/bioinformatics/bts635

65. R Core Team. R: A Language and Environment for Statistical Computing. Vienna, Austria: R Foundation for Statistical Computing; 2013. Available: www.R-project.org

66. Lun ATL, Riesenfeld S, Andrews T, Dao TP, Gomes T, Marioni JC. EmptyDrops: Distinguishing cells from empty droplets in droplet-based single-cell RNA sequencing data. Genome Biology. 2019;20: 63. doi:10.1186/s13059-019-1662-y

67. Zappia L, Oshlack A. Clustering trees: a visualization for evaluating clusterings at multiple resolutions. GigaScience. 2018;7. doi:10.1093/gigascience/giy083

Supporting information Captions

S1 Table. Illumina sequencing metrics.

S2 Table. Alignment statistics for *P. vivax* sporozoite scRNA-seq data.

S3 Table. Comparison of *P. vivax* sporozoite gene metrics with- and without-UTR information across each replicate

S4 Table. Overlap across datasets of additional genes detected in *P. vivax* sporozoites with UTR information and total counts in each replicate

S5 Table. Marker genes in *P. vivax* sporozoites across clusters

S6 Table. Genes identified as differentially expressed across pseudotime with corresponding summary statistics.

S7 Table. Summary of orthologous genes used in cross-species integrated analysis.

S8 Table. Conserved markers across *P. vivax* and *P. falciparum.*

S9 Table. Overlapping and species-specific genes detected in ORTHO clusters.

S10 Table. Genes differentially expressed between *P. vivax* and *P. falciparum* in each cluster.

S11 Table. Genes differentially expressed between sporozoites in clusters ORTHO_C3 and ORTHO_C4. Postive avg_log2FC: greater expression in ORTHO_C3.

S12 Table. Alignment statistics for *P. vivax* blood-stage scRNA-seq data downloaded from SRA (PRJNA603327) and remapped using STARsolo.

S13 Table. Differential gene expression between *P. vivax* sporozoites and erythrocytic-stage parasites.

Supplementary Figure 1. Related to Figure 1. Strategy used to assess *P. vivax* sporozoite transcriptomes at single-cell resolution.

Supplementary Figure 2. Related to Figure 1. Strategy used to assess *P. vivax* sporozoite transcriptomes at single-cell resolution.

Supplementary Figure 3. Related to Figure 2. Analysis of *P. vivax* sporozoite gene expression at single-cell resolution

Supplementary Figure 4. Related to Figure 3. Clustering and differential expression analysis of *P. vivax* sporozoites.

Supplementary Figure 5. Related to Figure 5. Integration of *P. vivax* and *P. falciparum* sporozoite datasets.

Supplementary Figure 6. Related to Figure 5. Integration of *P. vivax* and *P. falciparum* sporozoite datasets.

Supplementary Figure 7. Related to Figure 6. Integration and comparative analyses of *P. vivax* sporozoite and blood-stage parasite transcriptomes.

Figure

Click here to access/download;Figure;PvSpz_Figures_20211217.pdf

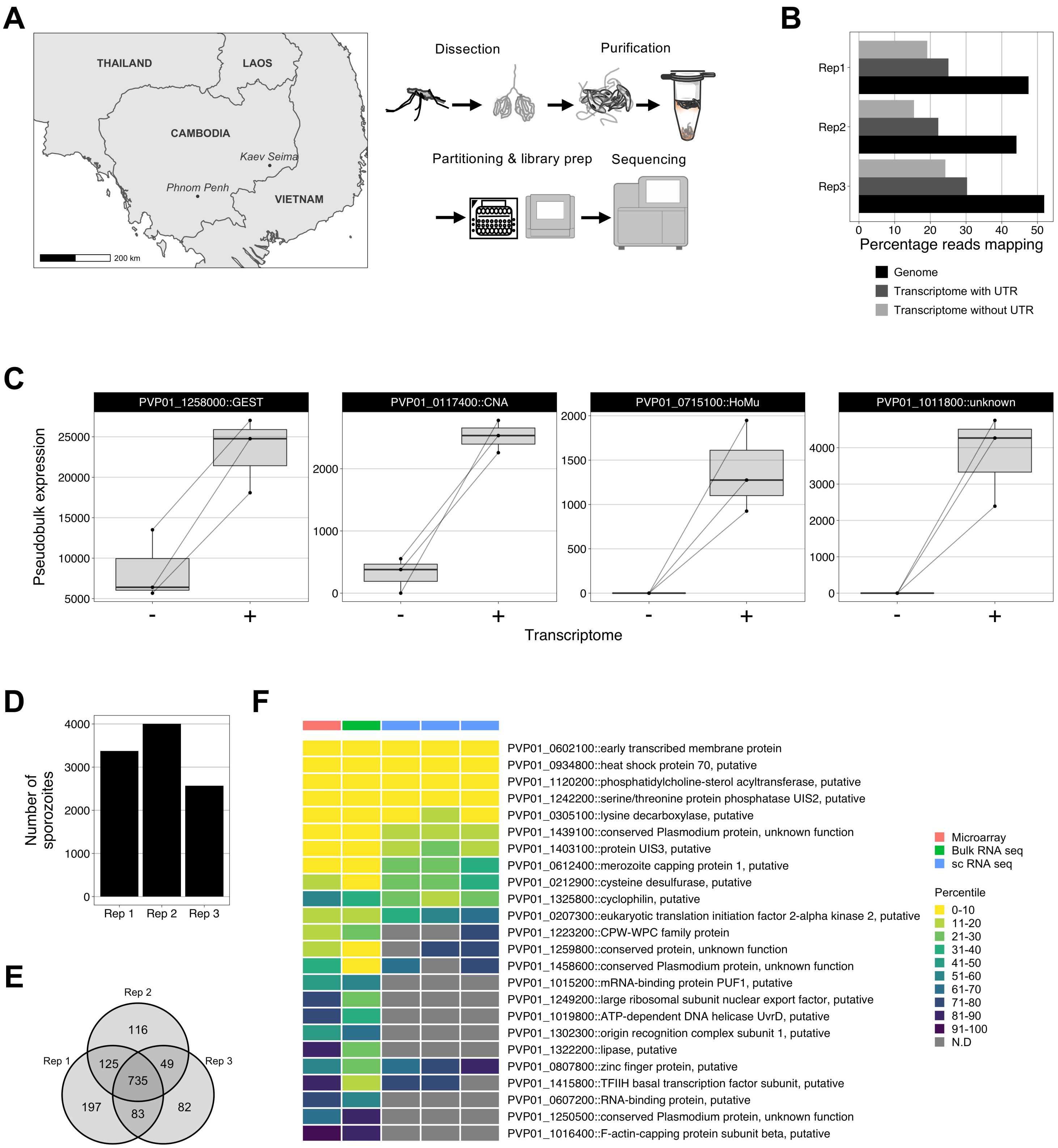

Figure 1. Strategy used to assess *P. vivax* sporozoite transcriptomes at single-cell resolution.
(A) Schematic illustrating the geographical region, sample preparation and sequencing pipeline used to capture single-cell transcriptomes of *P. vivax* sporozoites. (B) Percentage of reads aligning to the *P. vivax* P01 genome and transcriptome (with- or without- UTR information) across the three replicates. (C) 'Pseudobulk' visualisation of UMI counts aligned to *P. vivax* P01 transcriptome without (-) or with (+) UTR information. (D) Number of sporozoite transcriptomes retained post cell- and gene- filtering. (E) Number of unique and overlapping genes detected across scRNA-seq datasets.(F) Comparison of orthologous up-regulted in infective sporozoites (UIS) genes (obtained from *P.berghei)* across three high-throughput sequencing technologies (microarray: Westenberger *et al.* 2009, bulk-RNA seq: Muller *et al.* 2019 and single-cell RNA seq; current study). To account for the differences in the total number of genes detected across technologies, gene expression values were compared using percentile ranks. *GEST:* gamete egress and sporozoite traversal; *CNA:* serine/threonin protein phosphatase 2B catalytic subunit A; *HoMu:* RNA-binding protein Musashi; UTR: untranslated region.

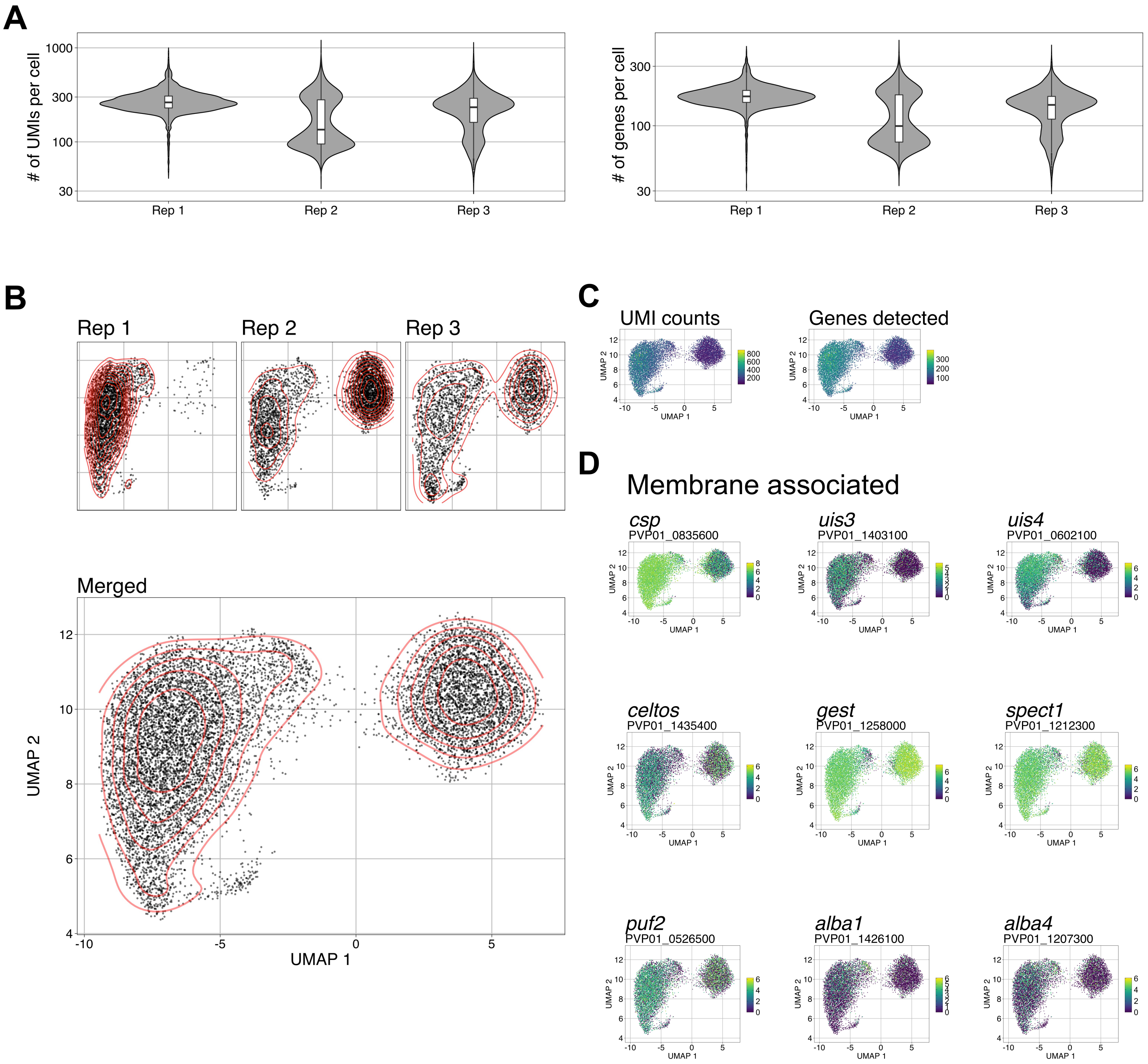

Figure 2. Analysis of *P. vivax* sporozoite gene expression at single-cell resolution.
(A) Violin plots (with boxplox overlaid) showing the distribution of unique molecular identifiers (UMIs) per cell (left), and genes detected be cell (right). (B) UMAPs of *P. vivax* sporozoite transcriptomes, visualized per replicate (upper) and combined (lower). Red-lines represent the density of cells represented in low dimensional space. (C) UMAPs of *P. vivax* transcriptomes colored by total number of UMIs (left) and total number of genes detected (right) per cell. (D) UMAPs of *P. vivax* sporozoite transcriptomes. Cells colored by expression of various genes implicated in sporozoite biology. Scale bar: normalised expression. *csp:* circumsporozoite protein; *uis3:* upregulated-in-infective sporozoites 3; *uis4*: upregulated-in-infective sporozoites 4; *celtos:* cell-traversal protein for ookinetes and sporozoites; *gest:* gamete egress and sporozoite traversal; *spect1*: sporozoite microneme protein essential for cell traversal 1; *puf2:* mRNA-binding protein PUF2; *alba1:* DNA/RNA binding protein Alba1; *alba4:* DNA/RNA binding protein alba4.

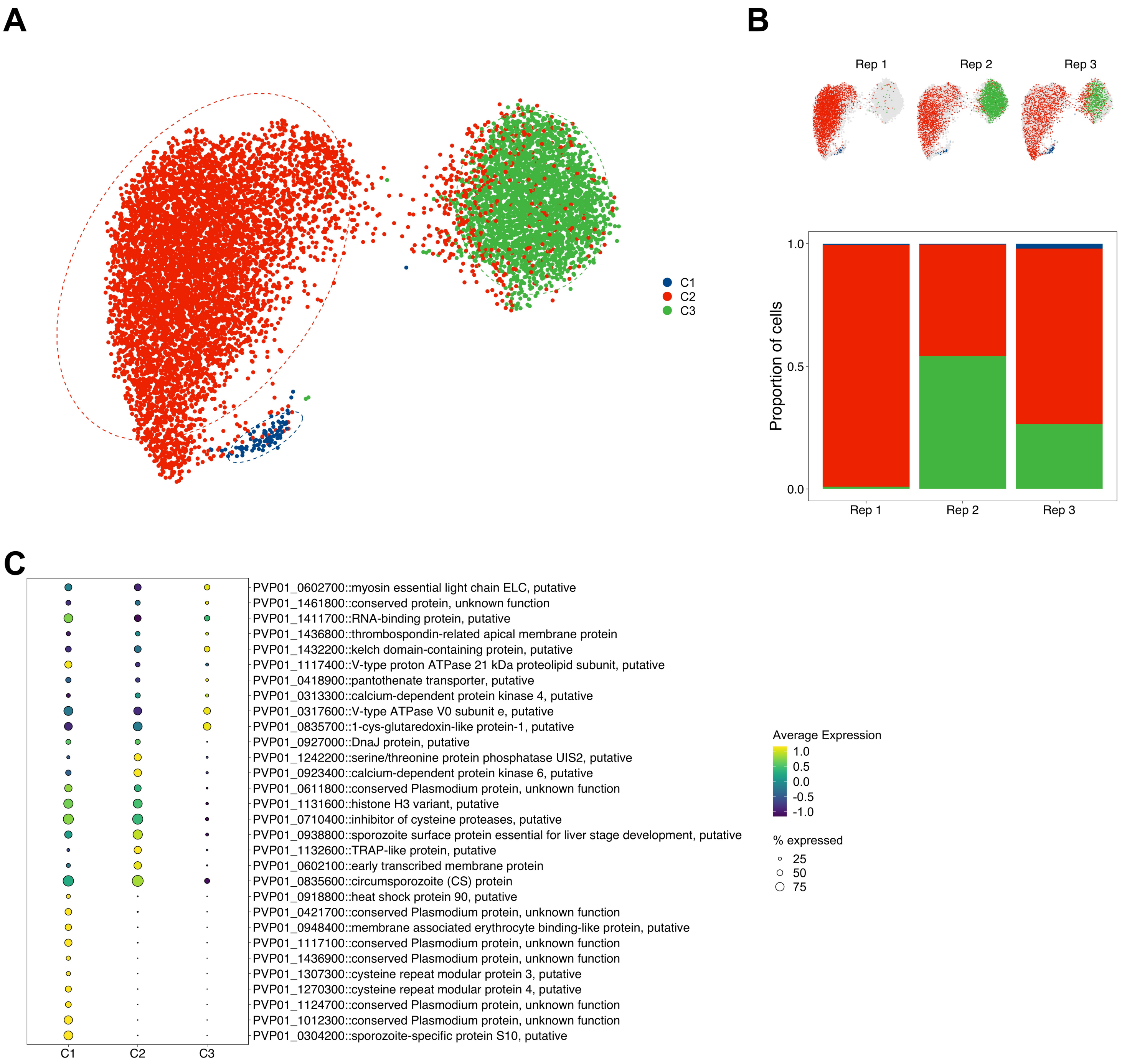

Figure 3. Clustering and differential gene expression analysis of *P. vivax* sporozoites.
(A) UMAP of integrated *P. vivax* sporozoite transcriptomes coloured by cluster (Leiden algorithm; resolution parameter = 0.1). (B) UMAP of integrated *P. vivax* sporozoite transcriptomes split by replicate (upper) and the percentage of sporozoites in each cluster from the replicate (lower). (C) Dot plot showing topmarkers that distinguish each of the three clusters. The size of the dot corresponds to the percentage of sporozoites expressing the gene. Scale bar: normalised expression, scaled.

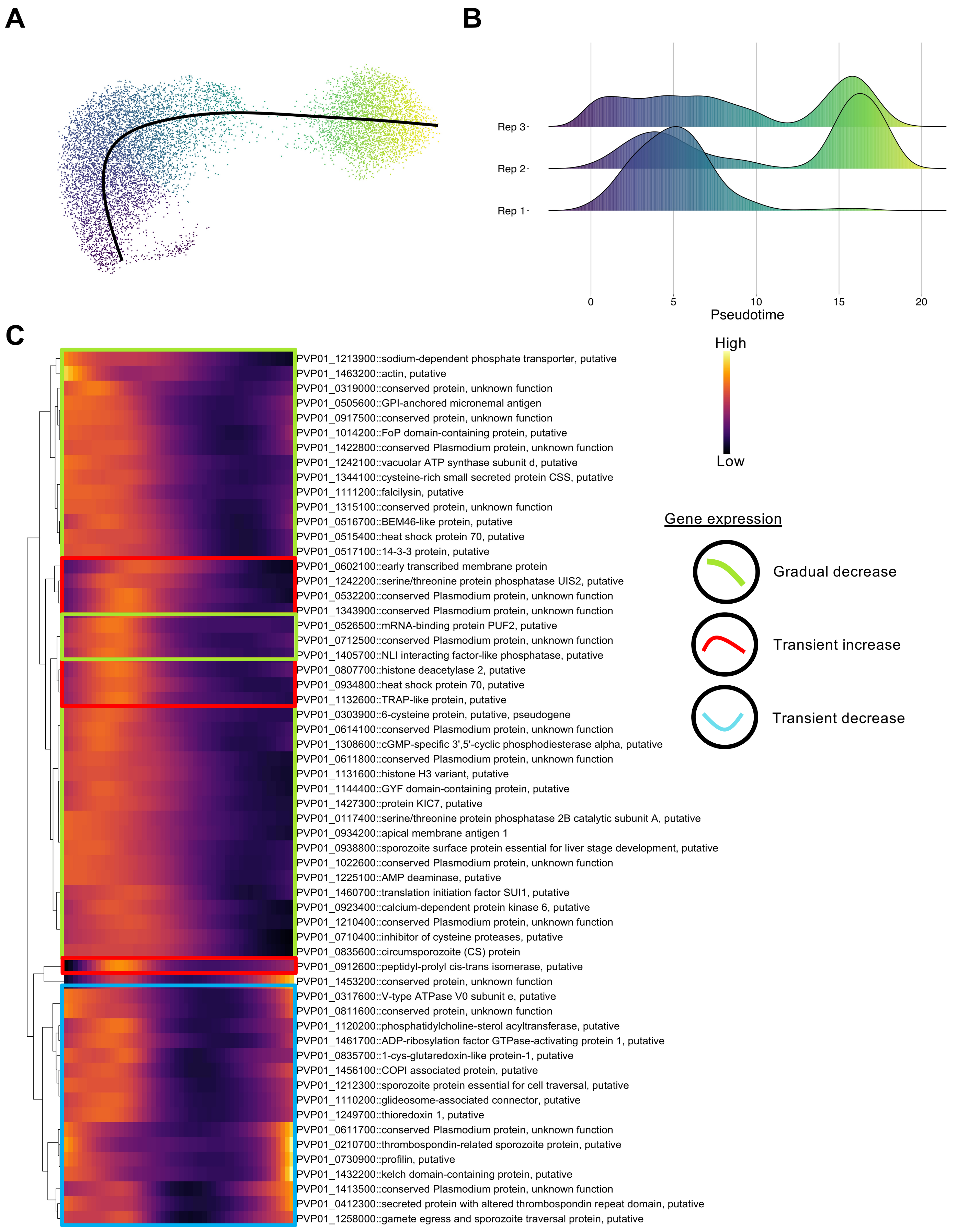

Figure 4. Pseudotime analysis of *P. vivax* sporozoites.
(A) UMAP *P. vivax* sporozoite transcriptomes coloured by progression along pseudotime. Colour scale matches pseudotime in panel B; where darker cells are representative of cells placed earlier in pseudotime and lighter cells are projected later along the pseudotime trajectory. (B) Distribution of cells along pseudotime faceted by replicate. (C) Heatmap showing genes differentially express across pseudotime (Data shown: mean pseudobulk >2000, waldStat >200 and FDR < 0.01). Genes are hierarchically clustered based on their pseudotime expression profile. Scale bar: normalized expression.

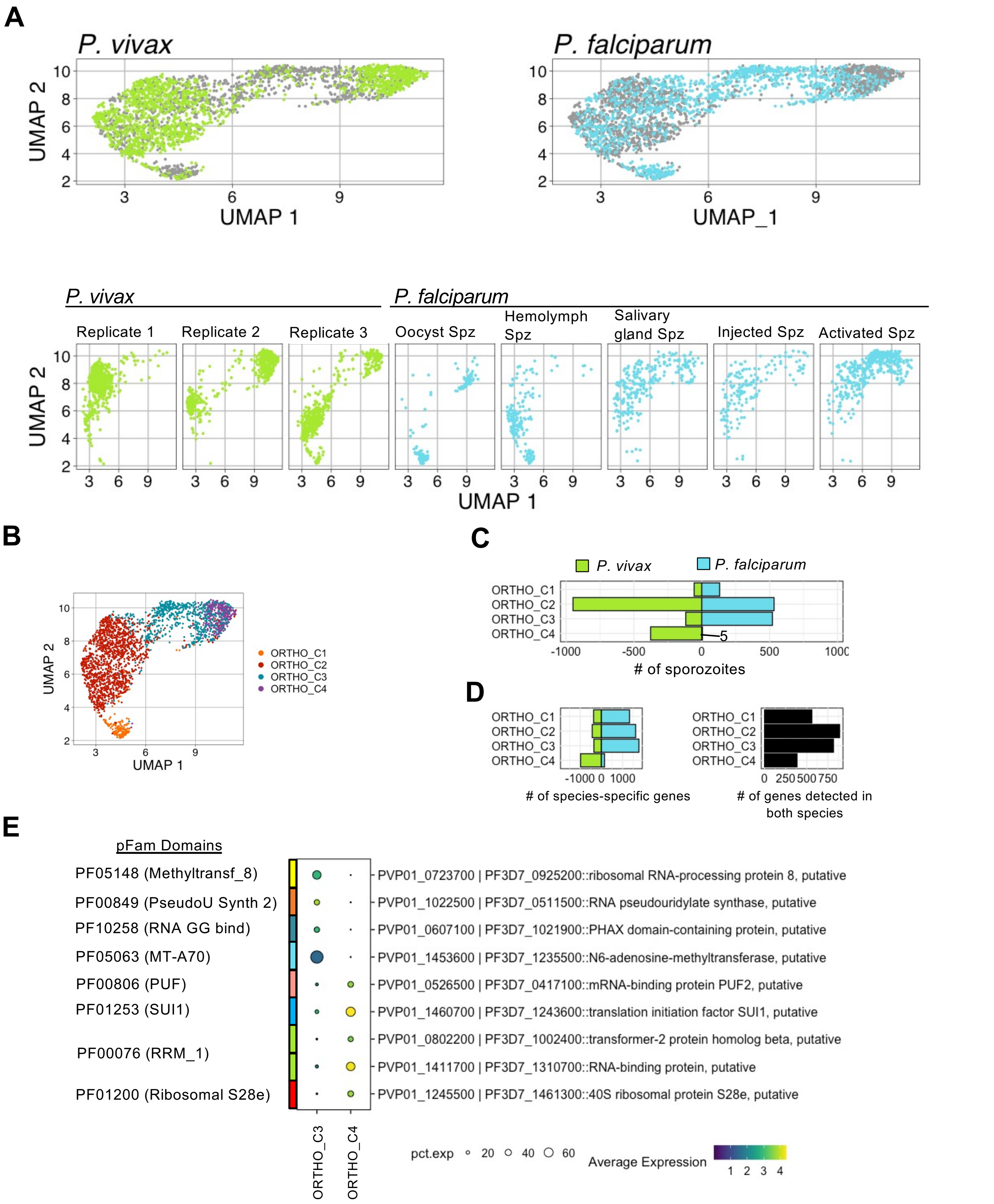

Figure 5. Integration of *P. vivax* and *P. falciparum* sporozoite datasets.
(A) UMAPs of integrated *P. vivax* and *P. falciparum* data. Top: split and coloured by species. Bottom: split by replicate (*P.vivax*) or sporozoite status (*P. falciparum*). (B) UMAP of integrated *P. vivax* and *P. falciparum* data coloured by cluster. (C) Number of cells contributing to each cluster from *P. vivax* and *P. falciparum* samples. (D) Number of species-specific genes (left) and conserved one-to-one orthologs (right) detected in each cluster. (E) Differentially expressed genes between clusters ORTHO_C3 and ORTHO_C4 with known Pfam domains. . Scale bar: normalized expression.

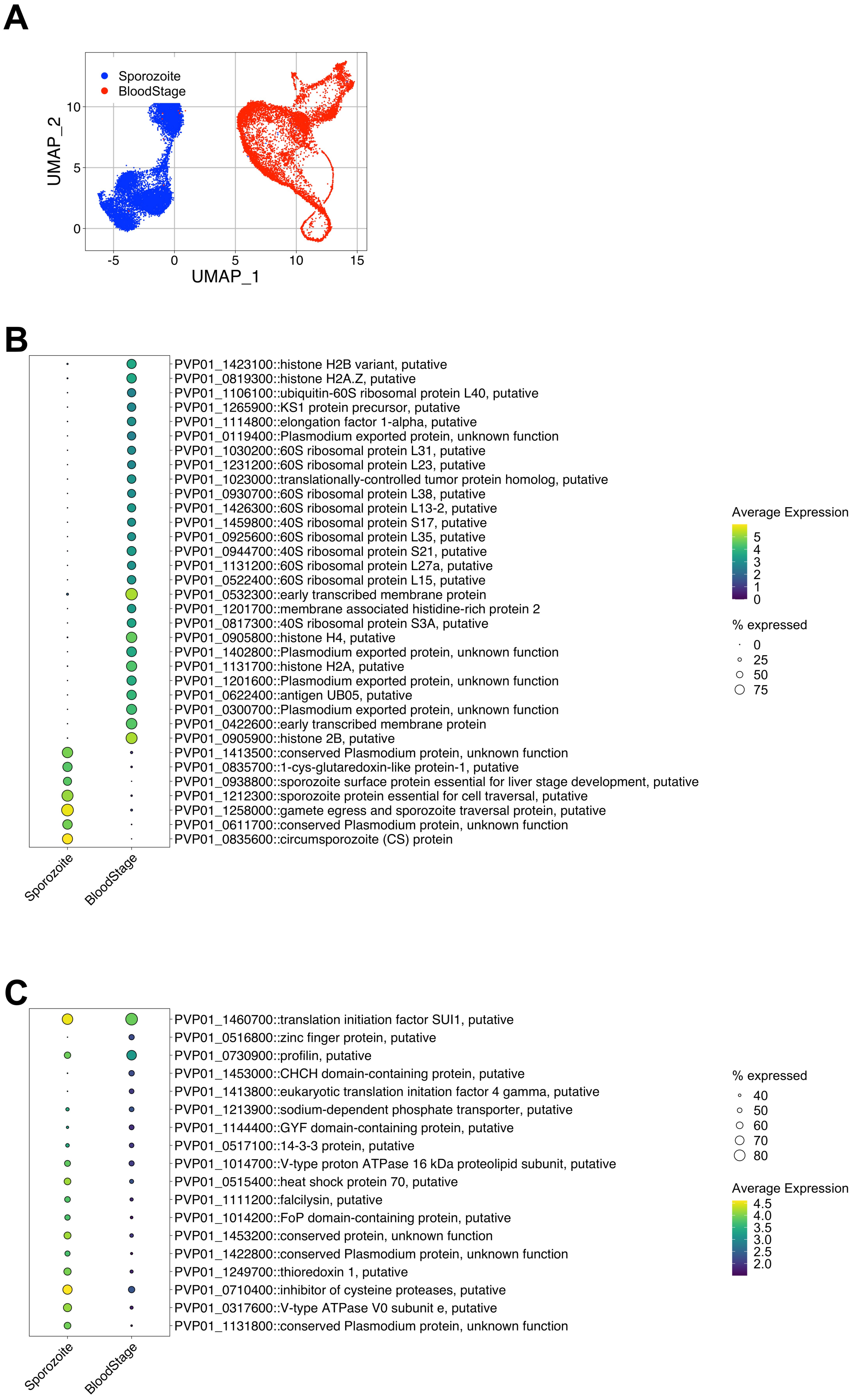

Figure 6. Integration and comparative analyses of *P. vivax* sporozoite and blood-stage parasite transcriptomes.
(A) UMAP of integrated *P. vivax* sporozoite and blood-stage parasite transcriptomic data. (B) Dot plot highlighting the top differentially expressed genes with stage-specific specific expression patterns (absolute difference in gene detection > 65% between sporozoites and blood stages). (C) Dot plot highlighting the top differentially expressed genes with detection in similar percentages of cells between sporozoites and blood-stage parasites. Scale bar: normalized expression.

Supporting Information Figures

Click here to access/download
Supporting Information
PvSpz_SupplementaryFigures_20211217.pdf

Supporting Information Tables

Click here to access/download
Supporting Information
PvSpz_SupplementaryTables_20211217.xlsx
