# Supplementary material for: Single-cell RNA sequencing of Plasmodium vivax sporozoites reveals stage- and species-specific transcriptomic signatures
Source: PLoS Negl Trop Dis. 2022 Aug 4;16(8):e0010633. doi: 10.1371/journal.pntd.0010633 (PMC9380936; doi:10.1371/journal.pntd.0010633)
Supplement: Attachment — Submitted filename: ResponseToReviewers.pdf [file pntd.0010633.s024.pdf]

Ivo Mueller, MSc, PhD
Professor and Co-Division Head
Population Health and Immunity Division
Walter and Eliza Hall Institute of Medical Research
1G Royal Parade, Parkville, Victoria 3052, Australia

Grouped response to reviewer's questions or suggestions:

Methods

Please note there is some discrepancy with track changes and line numbers. Line numbers are accurate when set to 'Simple Markup'. All changes are visible in 'All Markup'.

Regarding "Blood Samples, mosquitos and infections" and "Sporozoite isolations" (lines 388-414)

- Reviewer #2: A brief description of the blood samples collection protocol in line 389 should be provided.

- Reviewer #2: Could the number of patients used in this study be indicated? Was this blood pooled? Which protocol/criteria was used in screening and collection of blood samples

- Reviewer #1: Pg 13 lines 392-5. Please explain how mosquitoes containing oocyst were identified and kept/maintained to develop sporozoites.

We have provided additional clarification on the protocol used for *P. vivax* infected blood-collection and membrane feeding as well as identification of infected mosquitoes and the checking of midgut oocysts of *An. dirus* in sections "Blood samples, mosquitos and infections" at line 388. The text now reads:

> *Line 389-401: Blood samples from symptomatic patients with P. vivax presenting to local health facilities in Mondulkiri province (Kaev Seima) in Eastern Cambodia from September to November 2019 (Fig 1A) were collected via venipuncture into heparin-containing tubes. The blood was centrifuged for 5 minutes at 3000 rpm maintained at 37°C so that the serum be interchanged to that of a malaria-naïve individual with AB blood type (Interstate Blood Bank). Female Anopheles dirus colony aged 5-7 days in Mondulkiri were membrane fed, for one hour, the P. vivax blood-meal via custom-made insect membrane-feeders. Following the blood-meal, An. dirus mosquitoes were maintained at 26°C on a 12hr:12hr light:dark cycle and fed with 10% sucrose + 0.05% PABA solution. An. dirus from the feed were checked for P. vivax oocysts at six days post-feeding via midgut dissection and 1% Mercurochrome stain. If midguts were found to contain oocysts under a 20X microscope, the remaining An. dirus were transported to the Institut Pasteur of Cambodia Insectary Facility in Phnom Penh, Cambodia, where they were maintained under the same conditions described above.*

Ivo Mueller, MSc, PhD
Professor and Co-Division Head
Population Health and Immunity Division
Walter and Eliza Hall Institute of Medical Research
1G Royal Parade, Parkville, Victoria 3052, Australia

- Reviewer #1: Sporozoites were isolated 16-18 days after infectious blood meal. It is not clear how dissection was done and how the flies were treated before being dissected.

An. dirus dissections were aseptically performed by manual dissection by the expert dissection team to isolate the salivary glands that contain the *P. vivax* sporozoites used in this study into HBSS (as described at line 403-414). To further clarify this detail we have added to line 406-408:

> *Lines 403-408: P. vivax* sporozoites were isolated from the salivary glands of female *An. dirus* mosquitoes 16-18 days after their infectious blood-meal, where days post blood-meal was dependent on the replicate being processed. *An. dirus were immobilised with 70% Ethanol spray.* Each replicate represents one independent feed of a clinical isolate isolated from symptomatic *P. vivax* patients in Mondulkiri. A team of technicians performed aseptic salivary-gland dissections for a maximum period of one hour. Generally, ~75 to 100 mosquitoes were dissected in the one-hour sitting for each of the three replicates.

- Reviewer #3: What wasn't clear was the total number of unique parasite lines used in the initial mosquito infections and were these mixed infections or clonal.

In our study, the three replicates presented each represent an independent mosquito feed, each isolated from a different *P. vivax* patient in Mondulkiri. Given the practical limitations imposed when working with field isolates, we focused our studies on defining the transcriptomic signatures of the sporozoites and did not study the genetic background of each isolate. Each *Plasmodium* spp. infected blood-meal is species-typed using a real-time PCR assay, as previously described (Canier *et al.* 2013 *Mal Journal* 10.1186/1475-2875-12-405) to ensure no multi-species infections are processed. Determining the complexity of infection was not in our experimental plan as it has previously been shown that up to 92% of Cambodian *P. vivax* infections are polyclonal (Friedrich *et al.* 2016 *PLoS NTD* 10.1371/journal.pntd.0004526). We saw minimal batch effects between the three replicates and used current best-practices for batch-correction. We suggest that resolving the effect of *P. vivax* genetic diversity and transcriptomic signatures be a question of future scientific endeavours, however was beyond the scope of the current study.

To clarify the total number of *P. vivax* isolates used in our experimental design, we have added the following at line 406-407:

> *Each replicate represents one independent feed of a clinical isolated from symptomatic P. vivax patients in Mondulkiri.*

Ivo Mueller, MSc, PhD
Professor and Co-Division Head
Population Health and Immunity Division
Walter and Eliza Hall Institute of Medical Research
1G Royal Parade, Parkville, Victoria 3052, Australia

- Reviewer #3: It would be interesting if they could also consider other data sets e.g. the Vivax Sporozoite Consortium 2019 paper on salivary glad sporozoites or the Cubi et al., 2017 paper on P. cynomolgy hypnozoites to increase the power of finding the mechanisms or signatures of commitment to maintain persistent liver infection that may occur during salivary glad stage.

We thank the reviewer for this suggestion and have subsequently added Supplementary Table 5 (and previous Supplementary Table numbers have been adjusted accordingly throughout the text) as a comparison of the gene expression detected in the Thai *P. vivax* sporozoites studied by the Vivax Sporozoite Consortium 2019 *IJP* in terms of decile and rank of expression. This will allow readers to easily explore possible genes of interest identified in the two studies. We have also added additional columns to Supplementary Table 12 to include differential expression categorisation according to published literature (and as analysed in their publication and available in supplementary tables) for Gural *et al.* 2018, a bulk-seq differential expression study of hypnozoites and mixed-liver stages; Roth *et al.* 2018, a bulk-seq differential expression study of sporozoites in different media; and the differential expression of *P. vivax* sporozoites profiled in Jex *et al.* 2019, comparing sporozoites to blood-stage forms. We chose to limit these comparisons only to *P. vivax* available data as opposed to including orthologous comparison to *P. cynomolgi.* We have therefore included the following text in the methods at line 517-522:

> *Differentially expressed genes found in “hypnozoites” and “mixedLS” (adjusted p-value <0.01) from Gural et al. 2018 [68]; “MotilityActivation”, “LiverStageEarly” and “InsectStageFinal” sporozoites from Roth et al. 2018 [21]; and “sporozoite” and “blood-stage” parasites from Vivax Sporozoite Consortium 2019 [19] were detailed in this comparison.*

We also draw the reviewer’s attention to Figure 1F, where we detailed expression of orthologous upregulated-in infectious sporozoite genes in both the Vivax Sporozoite Consortium 2019 *IJP* bulk-RNA seq study and Westenberger *et al.* 2010 *PLoS NTD* microarray study of *P. vivax* sporozoites.

Conclusions

- Reviewer #2: The closing statements can be better re-worded to capture the broad public health concern of P. vivax malaria control (IVM) beyond the molecular significance.

We thank the reviewer for allowing us the opportunity to refine our closing message as follows;

> *Line* 376-379: *It is indeed such knowledge that underpins the success in developing the tools and strategies that are needed to control and ultimately eliminate this globally distributed parasite species thereby freeing endemic countries from the heavy public health burden it exacts.*

WEHI
brighter together

Ivo Mueller, MSc, PhD
Professor and Co-Division Head
Population Health and Immunity Division
Walter and Eliza Hall Institute of Medical Research
1G Royal Parade, Parkville, Victoria 3052, Australia

Other modifications

- Reviewer #1: Pg 17 line 328. I think it should be 'transcriptional'

Modified at current line 327.

- Reviewer #1: Pg 15, line 280-2…consider revising, difficult to understand.

Modified at current lines 280-282 as follows to improve clarity;

> *These findings indicate that despite the uniform morphology and cellular invasion approach used between the species; the underlying transcriptomic signature of individual sporozoites differs between species.*

- Reviewer #1: Pg 12 lines 347. The sentence is too long consider shortening it to be clear. What does 'intriguing' mean in this case?

To improve clarity, this sentence now reads at lines 346-349:

> *Noting the role of translational repression in eukaryotic developmental fating via RNA-binding proteins such as PUF2 [57], it is intriguing to speculate on possible links between this population and the bradysporozoites hypothesized to become hypnozoites.*

We use the word "intriguing" to emphasise this is an important biological connection between the discussed bradysporozoite hypothesis and the known function of PUF2 in other organisms but to emphasise this is further speculation given the lack of definitive evidence in our current study.

- Reviewer #1: Pg 14 'Hepatocyte infection and liver stage assessment' What is the need of describing a procedure that was described elsewhere? Consider deleting the entire paragraph.

We have removed this paragraph and added an additional reference of the protocol (Maher, Vantaux *et al.* 2021 *Bio*-protocol doi:10.21769/BIOPROTOC.4253) at line 417.

- Reviewer #2: The legend labels for Fig S4 (A) should be increased

We have modified the labels of Fig S4A for increased size in text Fig S4A.

Reviewer #2 suggested some grammatical changes in the PDF attachment that we have modified at
Line 29: addition of '*and causing malaria'*

Ivo Mueller, MSc, PhD
Professor and Co-Division Head
Population Health and Immunity Division
Walter and Eliza Hall Institute of Medical Research
1G Royal Parade, Parkville, Victoria 3052, Australia

Line 30: addition of ‘*parasite’*
Line 31: addition of *‘malaria’*
Line 59: deletion of *‘clearly’*
Line 69: addition of *‘the malaria’*

Unprompted modifications:

Inclusion of all Figure and Supplementary Figure/Table legends and text at the end of the manuscript.

Line 49: reactivation changed to ‘*activation’*
Line 73: reactivating changed to ‘*activating’*
Line 81: reactivation changed to ‘*activation’*
Line 119: 10X to *10x Genomics’*
Line 298: 0.1 to 0.01
Line 307: reveals to *reveal*

Some changes of normalized to normalised*.*

Express changed to *expressed* in Figure 4C legend text.

Addition of the word “RNA binding protein” into Figure legend for Figure 5E which was omitted in the original version.

Supplementary Figure 5- change of Dotplot to *Dot plot.*

Inclusion of S5 Table heading: *Comparison of PvSpz 10x rank to Vivax Sporozoite Consortium 2019 IJP bulk RNA seq rank.*

Change of S12 Table heading: *Genes differentially expressed between sporozoites in clusters ORTHO_C3 and ORTHO_C4. Positive avg_log2FC: greater expression in ORTHO_C4 (P. vivax specific).*

Github repository link added and will be made public upon acceptance of manuscript. Zenodo repository created and will be published upon acceptance of manuscript 10.5281/zenodo.6474355.
