# Supplementary material for: Single-cell RNA sequencing of Plasmodium vivax sporozoites reveals stage- and species-specific transcriptomic signatures
Source: PLoS Negl Trop Dis. 2022 Aug 4;16(8):e0010633. doi: 10.1371/journal.pntd.0010633 (PMC9380936; doi:10.1371/journal.pntd.0010633)
Supplement: Attachment — Submitted filename: PLosNTD.docx [file pntd.0010633.s022.docx]

This manuscript PNTD-D-21-01772, Ruberto et al., reports on a single-cell RNA-seq transcriptome analysis of the sporozoites of P. vivax parasites obtained from infected salivary gland of mosquito, compares them with those obtained from infected liver cells. So far, although parasites in the mosquito salivary gland are essential in malaria transmission to the mammalian host, no gene expression study of P. vivax parasites in this part of the fly have been done and single cell level with only studies limited studies done on the parasite inhabiting the liver cells. In this context, the presented study has certainly an important merit toward enhancing the understanding the transmission biology of P. vivax and can lead to identification of novel drug target as well as vaccine and diagnostic candidate. Additionally, this study also contributes important literature to the malaria scientific community.

Here are some minor comments:

-Pg 17 line 328. I think it should be ‘transcriptional’

-Pg 15, line 280-2…consider revising, difficult to understand.

-Pg 12 lines 347. The sentence is too long consider shortening it to be clear. What does ‘intriguing’ mean in this case?

Methodology

-Pg 13 lines 392-5. Please explain how mosquitoes containing oocyst were identified and kept/maintained to develop sporozoites.

Sporozoites were isolated 16-18 days after infectious blood meal. It is not clear how dissection was done and how the flies were treated before being dissected.

-Pg 14 ‘Hepatocyte infection and liver stage assessment’ What is the need of describing a procedure that was described elsewhere? Consider deleting the entire paragraph.
